# Wnt1’s Differential Effects on Craniofacial Bone and Tooth Development

**DOI:** 10.1177/00220345251336191

**Published:** 2025-06-02

**Authors:** R. Mahmoud, A. Simon, J. Luther, J. Pothe, Y. Du, C. Nottmeier, E. Okine, S. Knauth, M.G. Lopez, E. Bockamp, J. Krivanek, A. LeBlanc, J. Helms, M. Amling, M. Kaucka, T. Schinke, T. Koehne, J. Petersen

**Affiliations:** 1 Department of Orthodontics, University of Leipzig Medical Center, Leipzig, Germany; 2 Department of Osteology and Biomechanics, University Medical Center Hamburg, Hamburg, Germany; 3 Division of Plastic and Reconstructive Surgery, Department of Surgery, Stanford School of Medicine, Stanford University, Palo Alto, CA, USA; 4 Institute for Translational Immunology and Research Center for Immunotherapy, University Medical Center, Johannes Gutenberg University, Mainz, Germany; 5 Department of Histology and Embryology, Faculty of Medicine, Masaryk University, Brno, Czech Republic; 6 Centre for Oral, Clinical & Translational Sciences, Faculty of Dentistry, Oral & Craniofacial Sciences, King’s College London, London, UK; 7 Max Planck Institute for Evolutionary Biology, Plön, Germany

**Keywords:** *Wnt1* activation, transgenic mouse model, osteopetrosis-like pathology, osteoanabolic, osteoclastogenesis, enamel

## Abstract

The development of craniofacial bones and teeth relies heavily on the Wnt signaling pathway, yet the specific mechanisms and Wnt variants involved remain under continual investigation. Using publicly available single-cell sequencing data from the mouse incisor, we reveal *Wnt1* expression across dental structures and investigate its role using a *Col1a1*-dependent *Wnt1* transgenic mouse model. Inducing *Wnt1* early on affects craniofacial bone without disturbing tooth development, but prolonged embryonic induction leads to postnatal mortality with osteopetrosis-like bone overgrowth and malformed teeth. While tooth formation was initially unaffected by postnatal *Wnt1* induction, prolonged activation impaired tooth root formation and odontoblast differentiation, resulting in shortened roots and thinner dentin. Three-dimensional micro–computed tomography quantification reveal that both embryonic and postnatal activation of *Wnt1* significantly increase neural crest–derived craniofacial bone volume, whereas mesenchymal-derived craniofacial bones are unaffected. Importantly, osteoclastogenesis is suppressed by *Wnt1* in a dose-dependent manner, revealed through bulk RNA sequencing and in vitro experiments. These findings emphasize the differential effects of *Wnt1* on bone development based on origin and highlight its role in modulating osteoclast activity, indicating broader implications for craniofacial development and potential therapeutic avenues.

## Introduction

Craniofacial development is a complex process involving the intricate interplay of various tissues, signaling pathways, and molecular mechanisms, one of which is the Wnt signaling pathway. The Wnt pathway is a fundamental and evolutionary conserved pathway that plays a crucial role in various developmental processes. It consists of 2 main branches, the canonical or Wnt/β-catenin–dependent pathway and the noncanonical or β-catenin–independent pathway. The latter can be divided into the planar cell polarity pathway and the Wnt/Ca2+ pathway (Komiya and Habas 2008).

The Wnt pathway is activated by a family of secreted glycoproteins encoded by wingless/Wnt genes. To date, 19 Wnt ligands have been identified in mice and humans (Buechling and Boutros 2011).

Traditionally, Wnts are known to primarily engage with Frizzled (Fzd) receptors and the co-receptors LRP5/6 (Ren et al. 2021). However, Wnts can also interact with ROR family proteins (ROR1 and ROR2) and RYK in noncanonical Wnt signaling (Green et al. 2014), PTK7 in planar cell polarity signaling (Berger et al. 2017), and MuSK in neuromuscular junction formation (Banerjee et al. 2011). Since the canonical Wnt pathway fulfills a key role in the development of neural crest cells (Wu et al. 2003), which later give rise to most of the facial mesenchyme and subsequently the craniofacial skeleton and dental mesenchyme (Achilleos and Trainor 2015), misregulation of the pathway has been linked to genetic syndromes with bone and tooth malformations and different syndromes. With regard to craniofacial bone development, the complexity is underscored by the different embryonic origins and ossification mechanisms (Wei et al. 2017). For example, inactivation of the Wnt signaling pathway leads to craniofacial defects (Ko and Sumner 2021). Furthermore, postnatal tooth development and homeostasis are also heavily dependent on Wnt signaling, with misregulation leading to periodontal ligament disturbance, ankylosis (Wu et al. 2019), and growth failure (Zhang et al. 2013). Furthermore, homozygous mutations of *Wnt3* lead to tetramelia syndrome (Niemann et al. 2004), mutations in *Wnt5a* to Robinow syndrome (Person et al. 2010), mutations in SOST to Gorlin syndrome (Gorlin et al. 2001), and mutations in *Wnt7a* to Fuhrmann syndrome (Woods et al. 2006). Meanwhile, *Wnt10a* mutations have been shown to affect bone and teeth formation such as odontoonychodermal dysplasia (Adaimy et al. 2007), Schopf–Schulz–Passarge syndrome (Bohring et al. 2009), and tooth agenesis (Kantaputra and Sripathomsawat 2011). Furthermore, mutations in *Wnt1* led to recessive osteogenesis imperfecta (Pyott et al. 2013) and early onset of osteoporosis (Laine et al. 2013), and they play an essential role in bone homeostasis (Joeng et al. 2017).

We recently identified *Wnt1* as a bone anabolic Wnt ligand in long bones (Luther et al. 2018) and in alveolar bone and cementum formation during tooth maintenance (Nottmeier et al. 2021).

However, despite these findings, the precise role of *Wnt1* in craniofacial bone development and tooth formation in the context of embryonic and early postnatal development remains to be elucidated. Especially because *Wnt1* is a key regulator of neural crest cells, which constitute most of the facial mesenchymal cells (Echelard et al. 1994), we expect *Wnt1* to fulfill an important role in craniofacial development. Using a published single-cell RNA sequencing dataset from the growing mouse incisor (Krivanek et al. 2020), we identified a subset of *Col1a1*-positive cells that co-expressed *Wnt1*. Therefore, to investigate the embryonic and postnatal roles of *Wnt1*, in this subset of cells that accounted for osteoblasts, osteocytes, odontoblasts, and cementoblasts, we used inducible *Wnt1*-transgenic mice under the influence of the 2.3kb *Col1a1* fragment promoter. Embryos and pups were analyzed at E15, 5, P0, P14, and P28 to reveal the functions of *Wnt1* in craniofacial bone and tooth development. Inducing Wnt1 expression in Col1a1-expressing cells enables us to evaluate the potency of Wnt1 in mesenchymal cells, offering insights into its therapeutic potential in craniofacial bone and tooth development.

## Methods

Methods are described in the appendix. Animal experiments were conducted in accordance with the ARRIVE2.0 guidelines.

## Results

### Short-Term *Wnt1* Induction during Embryonic Development Affects Craniofacial Bone but Not Tooth Development

**Figure 1.**
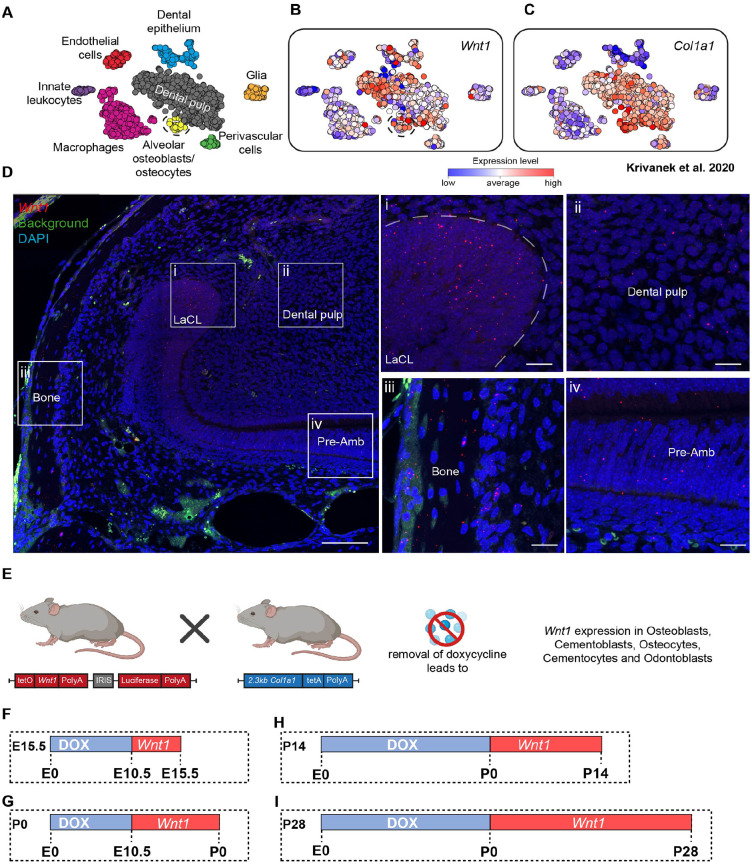
*Wnt1* expression in the murine incisor and experimental overview. (**A**) Published single-cell RNA sequencing dataset generated from the murine incisor mesenchyme highlighted different dental cell clusters. In the pagoda plot, blue indicates below-average expression, white represents the baseline, and red shows above-average expression, with the baseline defined as the average expression of the particular gene across all cells (modified from Krivanek et al. 2020). Individual cell transcriptomes were captured with the Smart-seq2 protocol to obtain high sequencing depth. Clustering using PAGODA revealed 17 major cell subpopulations, including the major immune, epithelial, and mesenchymal compartments. Validations and mapping of unbiasedly identified populations was based on the expression of selected marker genes in Krivanek et al. (2020). (**B**) Expression of Wnt1 in these cell clusters. (**C**) Expression of Col1a1 in the same cell clusters, created using the following published analysis: http://pklab.med.harvard.edu/cgi-bin/R/rook/tooth.general1/index.html. (**D**) Wnt1 expression in the region surrounding the cervical loop of an adult mouse incisor was assessed using RNA-Scope. LaCL, labial cervical loop; preAm, preameloblasts scale bar overview = 100 µm scale bar zoom inset = 20 µm. (**E**) Schematic presentation of the mating conditions and Wnt1 activation in transgenic mice (created in BioRender; https://BioRender.com/x42i303). (**F–I**) Schematic representation of the feeding regime used in this article. (**F**) E10.5 mouse embryos were deprived of doxycycline to induce the expression of Wnt1 until E15.5 (details are presented in Appendix Fig. 3). (**G**) E10.5 mouse embryos were deprived of doxycycline to induce the expression of Wnt1 until birth, where P0 pups were analyzed (details are presented in Fig. 2). (**H**) Schematic representation of the feeding regime showing the induced activation of Wnt1 at P0 by depriving doxycycline until mice were sacrificed at day 14 (details are presented in Appendix Fig. 8). (**I**) Schematic representation of the feeding regime showing the induced activation of Wnt1 at P0 by depriving doxycycline until mice were sacrificed at day 28 (details are presented in Fig. 3).

Analysis of a publicly available single-cell RNA sequencing dataset generated from the cervical loop from an adult murine incisor highlighted that *Wnt1* expression is present throughout the dental pulp, dental epithelium, alveolar osteocytes, and osteoblasts (Fig. 1A–C). This expression was confirmed by in situ hybridization for Wnt1 in the adult mouse incisor (Fig. 1D). The mouse incisor combines features of the cap stage (stem cell maintenance) and bell stage (cellular differentiation) while continuously cycling through aspects of these stages due to its uninterrupted growth. To investigate the role of *Wnt1* in this osteocyte and osteoblast subset, we used an inducible *Wnt1* transgenic mouse model (hereafter called Wnt1Tg), which included a doxycycline-dependent Tet-off system (Fig. 1E–I).

To study the effects of *Wnt1* activation during embryonic bone and teeth formation, we first examined the individual stages of wild-type development using micro–computed tomography (micro-CT). Phosphotungstic acid was used to stain soft tissues, enabling 3-dimensional (3D) segmentation of the mandibular bone and dental primordia (Appendix Fig. 1). Next, we used Wnt1Tg mice to investigate the effects of *Wnt1* in *Col1a1*-expressing cells during embryonic development. When *Wnt1* was initiated on the day of mating, no transgenic offspring were born (Appendix Fig. 2). Since *Wnt1* plays a fundamental role during neural crest migration (Ikeya et al. 1997), these results were anticipated. We then induced *Wnt1* at day E10.5, when neural crest cell migration is complete (Serbedzija et al. 1992), and analyzed transgenic mice at E15.5 (Appendix Fig. 3A–F), the stage when tooth development can be first well distinguished.

Micro-CT with phosphotungstic acid contrasting was used to analyze mandibular bone and incisors in E15.5 Wnt1Tg and control embryos. While the shape of the mandibular bone remained unchanged (Appendix Fig. 3A–B), its volume and thickness increased significantly (Appendix Fig. 3D, and Fig. 4C and E). Dental primordia of the lower incisor and molar appeared normal, and incisor volume showed no significant difference (Appendix Fig. 4F). These findings highlight *Wnt1* as a bone-anabolic ligand during early embryonic development without affecting initial tooth development.

### Wnt1 Induction at E10.5 Alters Craniofacial Bone and Tooth Morphogenesis by P0 and Leads to Postnatal Lethality

**Figure 2.**
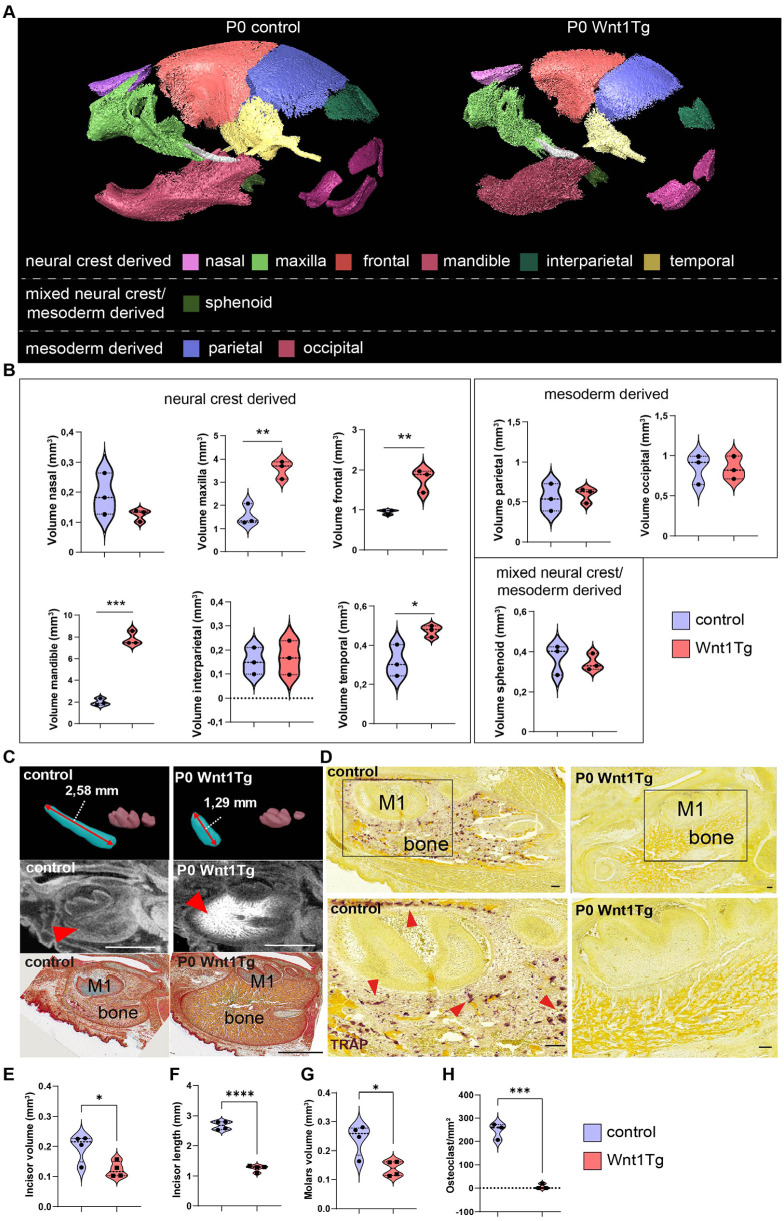
Bone and tooth malformations in P0 Wnt1Tg mice following 8 days of Wnt1 activation during embryonic development. (**A**) Three-dimensional (3D) segmentation of all major craniofacial bones of P0 Wnt1Tg pups and control skulls. (**B**) Quantification of all major neural crest–, mesoderm-, and mixed-derived bone. (**C**) Upper panel: 3D segmentation of mandibular molars and incisor of P0 Wnt1 pups and control. Middle panel: Micro–computed tomography (micro-CT) sagittal sections and (lower panel) pentachrome staining of P0 pups showing excessive bone formation (red arrow). Scale bar = 1 mm. (**D**) Representative images of mandibula tartrate-resistant acid phosphatase (TRAP) staining in P0 Wnt1Tg and control (lower panels) show higher magnification of the regions marked by the black rectangles. Scale bar = 100 µm. (**E–H**) Micro-CT quantification of (**E**) incisor volume, (**F**) incisor length, (**G**) molar volume, and (**H**) osteoclast quantification in TRAP staining in control and P0 Wnt1Tg pups. *n* = 4, except for B and H: *n* = 3. *P < 0.05, **P < 0.01, ***P < 0.001.

Next, we evaluated later stages of tooth development to determine if they were affected by *Wnt1* activation (Fig. 2A and Appendix Fig. 4). To our surprise, *Wnt1* activation starting at E10.5 resulted in P0 pups dying shortly after birth. In-depth analysis using micro-CT coupled with histology revealed an altered skull shape in P0 Wnt1Tg mice with a recessed lower lip and a steep eruption angle of the lower incisors (Appendix Fig. 4A). Further analysis of the mandible length and height revealed a shorter mandible in P0 Wnt1Tg mice compared with controls (Appendix Fig. 4B). Nevertheless, bone volume was increased in most of the neural crest–derived bones, including the mandible, maxilla, and frontal bone (Fig. 2A and B and Appendix Fig. 5A). Interestingly, mesoderm-derived bones such as the parietal and occipital bone exhibited no increase in bone volume (Fig. 1A and B). However, in situ hybridization of *Wnt1* and *Col1a1* revealed that all major bones (neural crest– and mesoderm-derived bones) express *Wnt1* and *Col1a1* (Appendix Fig. 6). In terms of teeth, the lower incisor was significantly shortened compared with controls (Fig. 2C and F). Histology analysis revealed that odontoblasts were altered in Wnt1Tg mice (Appendix Fig. 7C and D). Despite no increased expression of Wnt1 in ameloblasts in Wnt1tg mice (Appendix Fig. 7E), enamel of the incisor was missing (Appendix Fig. 7F and G). When investigating the molars, a similar but reduced phenotype was detected. This was manifested in later erupting M2 molars and M1 cusps, where the volume of Wnt1Tg molars was significantly decreased (Fig. 2C and G). In addition, tartrate-resistant acid phosphatase (TRAP) staining revealed a severe inhibition of osteoclastogenesis in P0 Wnt1Tg compared with controls (Fig. 2D and H and Appendix 7A and B).

### Postnatal Overexpression of *Wnt1* Affects Tooth Roots and Surrounding Bone Structures but Not Crown Formation

**Figure 3.**
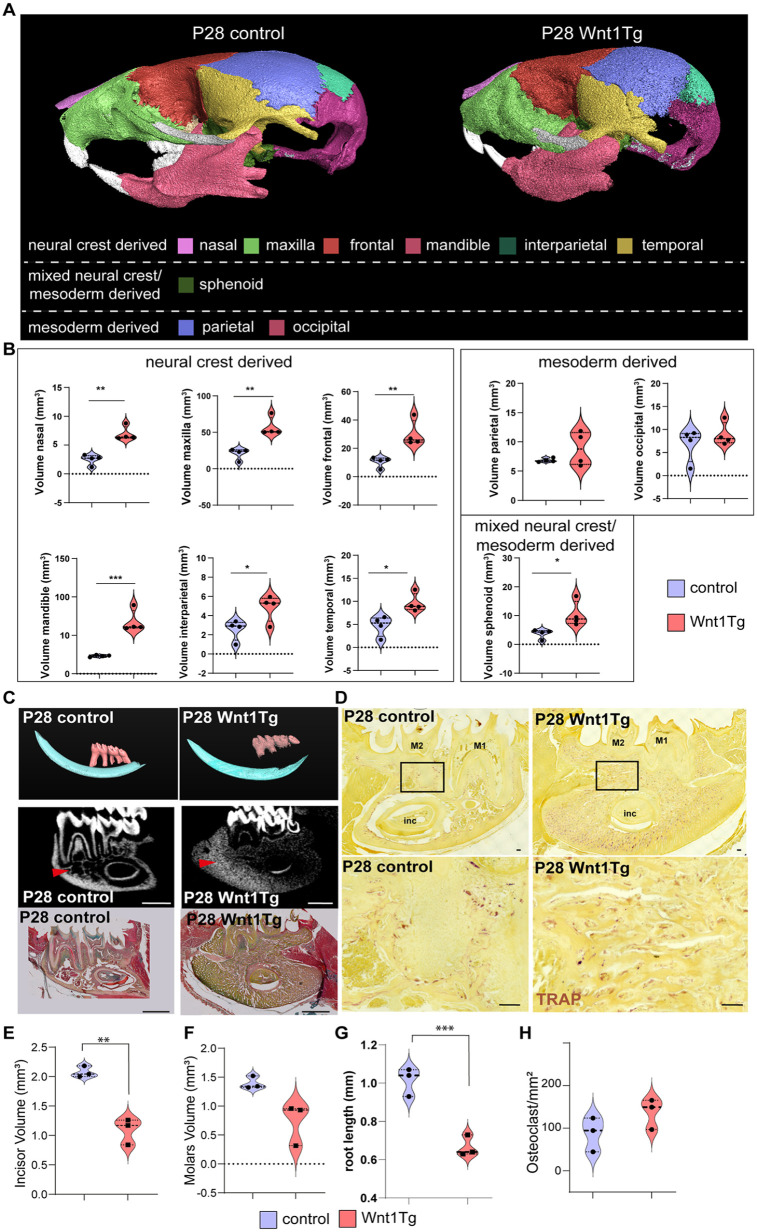
Long activation of Wnt1 after birth leads to craniofacial bone and tooth root alterations. (**A**) Three-dimensional (3D) segmentation of all major craniofacial bones of P28 Wnt1Tg pups and control skulls. (**B**) Quantification of all major neural crest–, mesoderm-, and mixed-derived bone. (**C**) Upper panel: 3D segmentation of mandibular molars and incisor of P28 Wnt1Tg pups and control. Middle panel: Micro–computed tomography (micro-CT) sagittal sections and (lower panel) pentachrome staining of P28 pups showing excessive bone formation (red arrow). Scale bar = 1 mm. (**D**) Representative images of mandibular tartrate-resistant acid phosphatase (TRAP) staining in P28 Wnt1Tg and control (lower panels) show higher magnification of the regions marked by the black rectangles. Scale bar = 100 µm. (**E–H**) Micro-CT quantification of (**E**) incisor volume, (**F**) molar volume, (**G**) root length, and (**H**) osteoclast quantification in TRAP staining in control and P28 Wnt1Tg pups. *n* = 4, except for E and H: *n* = 3, *P < 0.05, **P < 0.01, ***P < 0.001.

To investigate the specific effect of *Wnt1* activation during the early phase of craniofacial development, postnatal activation (starting at P0) of *Wnt1* was initiated and mice were sacrificed at P14 or P28 (Appendix Fig. 8 and Fig. 3). Analysis of the skull wall thickness of P14 and P28 animals revealed thickened neural crest–derived bones in Wnt1Tg mice compared with controls (Appendix Fig. 5B and C), which was confirmed using volume measurements of all major neural crest– and mesoderm-derived bones (Appendix Fig. 8A and B and Fig. 3A and B). At P14, 3D segmentation of the molars and incisors coupled with pentachrome staining confirmed the excessive bone formation with no alteration in odontoblasts or ameloblasts (Appendix Fi. 8C and Appendix Fig. 9A and B). Trabecular bone of the mandible increased significantly, while cortical bone remained unaffected (Appendix Fig. 9C and E). Remarkably, the increase in bone mass after 14 d was minor compared with 8 d induced P0 pups (Appendix Fig. 5A and B). Contrary to our observation in P0 pups, quantification of TRAP staining showed no inhibition of osteoclastogenesis in P14 Wnt1Tg versus control (Appendix Fig. 8D and G).

At P28, molars showed no significant difference in volume, but molar roots were significantly shorter (Fig. 3C–G). Pentachrome staining of the first molar revealed differences in tooth morphology and ectopic pulp calcification and significantly thinner dentin (Appendix Fig. 10A and B). Trabecular bone of the mandible increased significantly, while cortical bone remained unaffected (Appendix Fig. 10C–E). TRAP staining revealed that *Wnt1* activation did not exert an overall suppressive effect on osteoclast formation (Fig. 3D). Quantification across the entire mandible did not show statistically significant differences in osteoclast numbers (Fig. 3H).

### Molecular Insights into *Wnt1* Activation and Its Effects on Bone and Odontogenesis at Key Postnatal Stages

**Figure 4.**
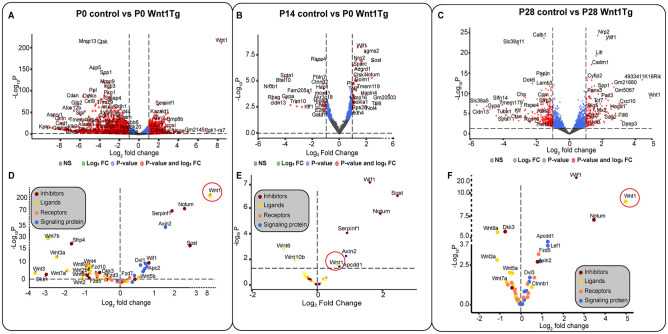
Molecular insights into *Wnt1* activation at developmental stages P0, P14, and P28. (**A**) Volcano plot showing differentially up- and downregulated genes with |log2fc| > 1 in P0 Wnt1Tg versus control. (**B**) Volcano plot showing differentially up- and downregulated genes with |log2fc| > 1 in P14 Wnt1Tg versus control. (**C**) Volcano plot showing differentially up- and downregulated genes with |log2fc| > 1 in P28 Wnt1Tg versus control. (**D–F**) Volcano plots showing ligands, receptors, signaling proteins, and inhibitors involved in the Wnt signaling pathway at developmental stages (**D**) P0, (**E**) P14, and (**F**) P28.

To understand the molecular differences, we performed RNAseq analysis of mandibles from P0, P14, and P28 Wnt1Tg and control mice (Fig. 4). At P0, osteoclast markers, such as *Ctsk* and *Acp5*, were significantly impaired alongside markers of bone ossification and remodeling such as *Spp1* and *Mmp13* (Fig. 4A). To compare the biological processes affected by *Wnt1* activation in P0 pups, we performed Gene Enrichment Ontology (GO) analysis (Appendix Fig. 11B and 12D). The results support our observation that *Wnt1* affects odontogenesis and bone mineralization.

Using Kyoto Encyclopedia of Genes and Genomes (KEGG) analysis, we identified altered pathways including the Wnt pathway, and osteoclast differentiation (Appendix Fig. 12C). A deeper analysis of the canonical Wnt pathway revealed an 8-fold increase in *Wnt1* expression, which overactivated downstream components such as *Axin2*, *Dvl1*, and *Dvl2* along with the suppression of most Wnt ligands and Wnt receptors (*Fzd8*, *Ror1*, *Fzd10*, *Fzd3*, *Musk*, *Ror2*, *Fzd5*) and an upregulation of Wnt signaling inhibitors and antagonists such as *Sost*, *Serpinf1*, and *Notum* (Appendix Fig. 11C–G and Appendix Fig. 12A). The osteoclast differentiation pathway was inhibited with a decrease in the expression of secreted *M-CSF* along with *Nfatc1*, *Rankl*, and osteoclast-specific proteins such as *Ctsk* and TRAP (Appendix Fig. 12B). These results support our histological observations indicating a strong suppression of osteoclasts.

RNAseq analysis from P14 Wnt1Tg and controls showed increased activity in Wnt pathway inhibitors (*Wif1*, *Tcf7*, *Sost*, *Notum*) and bone-related genes (*Ctsk*, *Sparc*, *Tmem119*, *Mmp16*) (Fig. 4B and Appendix Fig. 13).

GO analysis revealed that clusters related to bone mineralization, ossification, odontogenesis, osteoclast differentiation, bone remodeling, and resorption were affected (Appendix Fig. 13B and Appendix Fig. 14D). Furthermore, KEGG analysis highlighted fewer affected pathways than P0 Wnt1Tg, but the Wnt pathway was still significantly activated (Appendix Fig. 13C and D and Appendix Fig. 14A and C). Moreover, analysis of the osteoclast differentiation pathway indicated upregulation of *Nfatc1*, in addition to *Rankl* and *Opg* expression, accompanied by high expression of the osteoclast-specific proteins *Ctsk* and *Trap*; meanwhile, *M-CSF* levels remained unchanged (Appendix Fig. 14B).

RNAseq analysis from P28 Wnt1Tg and controls revealed upregulation of *Wif1*, *Lifr*, and *Panx3*. Meanwhile, *Sfrp2* was significantly downregulated (Fig. 4C). GO analysis indicated that the affected genes exhibited clusters resembling those found in P0 Wnt1Tg and P14 Wnt1Tg, focusing on bone mineralization, bone ossification, and odontogenesis (Appendix Fig. 15B, Appendix Fig. 16D). Moreover, KEGG analysis showed the same affected pathways as in P14 Wnt1Tg and P0 Wnt1Tg (Appendix Fig. 16C). Detailed analysis of the Wnt pathway in P28 Wnt1Tg revealed higher activation compared with P14 Wnt1Tg and lower activation compared with P0 Wnt1Tg (Fig. 4A–C). The heat map and volcano plot of the Wnt pathway components indicated upregulation of only *Serpinf1* and *Notum* in P28 Wnt1Tg, while *Sost* was not affected. Besides, 5 Wnt ligands were significantly downregulated (*Wnt3a*, *Wnt5a*, *Wnt7a*, *Wnt9a*, and *Wnt10b*); meanwhile, *Wnt1* was 5-fold upregulated (Appendix Fig. 15D–G and Appendix Fig. 16A). Additionally, the osteoclast differentiation pathway in P28 Wnt1Tg versus controls was more activated compared with P14 Wnt1Tg. This activation was manifested by increased expression of secreted *M-CSF*, *Rankl* along with its antagonist *Opg* and its receptor *Rank*, *Nfatc1*, and osteoclast-specific proteins such as *Ctsk* and *Trap* (Appendix Fig. 16B).

### *Wnt1* Inhibits Osteoclastogenesis in a Dose-Dependent Manner

**Figure 5.**
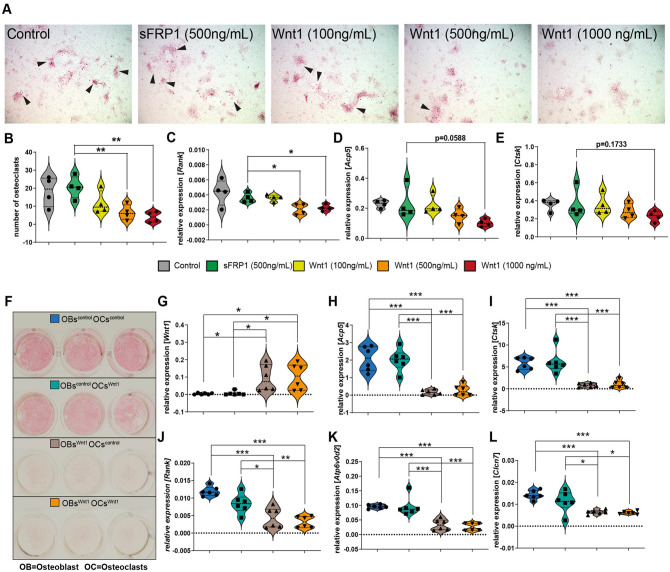
Effect of Wnt1 on osteoclast differentiation and gene expression in bone marrow cells and osteoblast/osteoclast co-cultures. (**A**) Tartrate-resistant acid phosphatase (TRAP) staining of isolated bone marrow cells incubated for 8 d with various concentrations of *Wnt1*/sFRP1 in combination with *M-CSF* and *Rankl*. (**B**) Quantification of the number of osteoclasts in each well. (**C**) Relative expression of *Rank* using quantitative polymerase chain reaction (qPCR). (**D**) Relative expression of Acp5 using qPCR. (**E**) Relative expression of *Ctsk* using qPCR. *n* = 4, *P < 0.05, **P < 0.01. (**F**) Representative images of TRAP staining from osteoblast/osteoclast co-culture experiments. (**G**) Relative expression of *Wnt1* using qPCR. (**H**) Relative expression of *Acp5* using qPCR. (**I**) Relative expression of *Ctsk* using qPCR. (**J**) Relative expression of *Rank* using qPCR. (**K**) Relative expression of *Atp6v0d2* using qPCR. (**L**) Relative expression of *Clcn7* using qPCR. *n* = 6, *P < 0.05, **P < 0.01.

To investigate whether the observed discrepancy in osteoclastogenesis between P0 and P14/P28 pups may stem from a dose-dependent influence, we conducted an in vitro osteoclastogenesis assay by differentiating bone marrow into osteoclasts. Bone marrow isolated from wild-type mice was stimulated with varying concentrations of Wnt1/sFRP1, alongside sFRP1 as control, assuming 1:1 complex formation ratio of *Wnt1* and sFRP1. Our findings revealed a significant inhibition of osteoclast formation with increasing *Wnt1* concentration (Fig. 5A and B). In addition, mRNA expression of *Rank* was significantly downregulated in higher *Wnt1* concentrations compared with the sFRP1 control (Fig. 5C).

Furthermore, to test whether *Wnt1*-expressing osteoblasts can inhibit osteoclasts, we conducted co-culture experiments to investigate this interaction. Bone marrow cells isolated from femora, tibiae, and pelvis of *Wnt1Tg* and control mice were plated in 24-well plates. Osteoblast differentiation was induced at confluency using osteogenic medium (details are given in the appendix). After 7 d of differentiation, freshly isolated bone marrow cells from *Wnt1Tg* and control mice were seeded on top of the differentiated osteoblasts. Osteoclast differentiation was then induced for 9 d (details are in the appendix). Cultures were analyzed via TRAP staining, which revealed a significant reduction in TRAP-positive multinucleated cells when *Wnt1Tg* osteoblasts were co-cultured with osteoclast precursors compared with controls (Fig. 5F–L). These findings demonstrate that *Wnt1*-expressing osteoblasts inhibit osteoclast formation, further supporting our hypothesis that *Wnt1* indirectly suppresses osteoclastogenesis through its effects on osteoblasts.

## Discussion

Using a transgenic mouse model, we investigated the effects of *Wnt1* induction at different developmental stages, employing various techniques to assess phenotypic changes. Our results indicate that *Wnt1* activation during early embryonic development (E10.5 to E15.5) did not disrupt tooth bell formation but significantly affected mandibular bone development. This finding is of interest given previous studies highlighting Wnt/β-catenin signaling’s role in tooth morphogenesis (Chen et al. 2009). Our findings show that *Wnt1* induction in *Col1a1*-expressing cells does not affect early tooth development but affects subsequent craniofacial bone formation.

### Bone Origin Determines Osteoanabolic Effects

Intriguingly, the phenotype of P0 pups differed drastically from P14 pups and revealed an osteopetrosis-like pathology characterized by a massive increase in bone volume, a severe decrease in osteoclasts, shortened teeth, and abnormal odontoblast formation. The observed tooth and enamel phenotype is likely secondary to the extensive bone growth, similar to the findings previously published on odontomas in osteopetrotic *Src^-/-^* mice (Koehne et al. 2013). Detailed investigation of the affected bones revealed that while the mandible, maxilla, and frontal bone responded strongly to *Wnt1* induction, other cranial bones remained relatively unaffected. Here, we noticed that the strongly affected bones ossify intramembranously (Gilbert 2000). In intramembranous bone, many osteoblasts are found early (Lana-Elola et al. 2007). Thus, it likely contributes to the constitutive activation of Wnt/β-catenin signaling and amplifies this effect through a positive feedback loop. This mechanism may explain the substantial increase in bone mass, with osteoblasts populating the endochondral-formed bone at a later stage (Ortega et al. 2004). However, this approach alone fails to account for the varying osteoanabolic effects seen in different bones. Especially, considering the parietal bone, an intramembranous ossification example, shows no response to *Wnt1* stimulation (Fig. 2, Appendix Fig. 8, and Fig. 3). This discrepancy may stem from differing embryonic origins of osteoblasts. While the osteoblasts of the mandible, maxilla, nasal bone, and frontal bone originate from the neural crest, those in the parietal bone derive from the mesoderm (Jin et al. 2016). This distinction significantly influences their osteogenic potential; neural crest–derived osteoblasts form bone more rapidly but exhibit lower differentiation propensity compared with mesoderm-derived parietal osteoblasts (Xu et al. 2007). Here, it would be of interest to investigate how these differences contribute to the varying osteoanabolic response to *Wnt1* stimulation.

### *Wnt1* Suppresses Osteoclastogenesis in a Dose-Dependent Manner

Another interesting finding of our study was the complete inhibition of osteoclastogenesis in *Wnt1*-induced P0 mandibles accompanied by high bone formation, whereas P14 pups (activated *Wnt1* for 14 d) or P28 pups (activated *Wnt1* for 28 d) displayed an elevated bone phenotype, without an observed effect on osteoclasts. These findings align with the existing literature documenting the varied effect of Wnt signaling on osteoclastogenesis.

For example, activation of *Wnt4* under the control of the 2.3kb *Col1a1* promoter led to osteoclast inhibition and prevented estrogen deficiency–induced osteoporosis in vivo (Yu et al. 2014). Furthermore, constant activation of β-catenin in differentiated osteoblasts also under the control of the *Col1a1* promoter inhibited osteoclastogenesis and increased bone formation (Glass et al. 2005). On the other hand, activation of *Wnt1* under the control of 2.3kb *Col1a1* promoter for 9 wk in adult mice resulted in no significant reduction in osteoclasts (Luther et al. 2018). Moreover, activating β-catenin in *Dmp1*-expressing osteoblasts and alveolar bone osteocytes resulted in significantly higher osteoclast and osteoblast activity compared with controls (Wu et al. 2019).

By using an in vitro bone marrow osteoclast differentiation assay, we revealed that *Wnt1* exhibits a dose-dependent inhibition effect on osteoclastogenesis. This aligns with our in vivo observations, which indicate a robust induction of *Wnt1* during early embryonic development, whereas the initiation of *Wnt1* postnatally is comparatively weaker. This dose-dependent effect can also explain the diverse and in part contradictory observations in the literature regarding the Wnt signaling impact on osteoclastogenesis, as previous studies have not investigated the extent of Wnt signaling activation. Furthermore, using the co-culture experiment, we identified that osteoblasts can influence osteoclastogenesis through secretion of Wnt ligands, thereby regulating Wnt signaling in osteoclasts. This aligns well with a study that showed how Wnt1 signaling regulates osteoclast differentiation in a juxtacrine manner (Wang et al. 2019).

In conclusion, our finding indicates that prolonged activation of *Wnt1* during development has detrimental effects on tooth morphogenesis. Furthermore, our study provides valuable insights into the dose-dependent and cell-specific effects of *Wnt1* signaling on craniofacial bone and tooth development. The findings contribute to our understanding of the intricate regulatory mechanisms involved in these processes. A limitation of the used Tet-off system is the potential gene expression during doxycycline administration. However, adult mice with continuous doxycycline exposure (Nottmeier et al. 2021) showed no bone or tooth phenotypes, minimizing concerns about unintended gene expression. Another limitation is a nonlinear dose response after doxycycline removal. It was therefore important to perform RNA sequencing of each analyzed stage and to interpret the results according to the respective *Wnt1* expression. Further research, including investigating the downstream targets and signaling interactions of *Wnt1*, will provide a more comprehensive understanding of the molecular mechanisms governing craniofacial development. Since the stimulatory effect of *Wnt1* on osteoblasts at all stages is undisputed, a focused pharmacologic strategy to activate osteoblasts while concurrently avoiding influence on osteoclasts holds significant promise as a therapeutic avenue for addressing bone metabolic disorders such as osteoporosis. For regenerative dentistry, in addition to the osteoanabolic effect, the positive effect on cementum formation in later developments as shown in Nottmeier et al. (2021) is also important, which makes *Wnt1* an interesting target for periodontology in the future. Therefore, deciphering the exact mechanisms behind the *Wnt1* signaling is essential and should remain the subject of future research to develop specifically effective pharmaceuticals. Furthermore, β-catenin can initiate signalling through its transcription-independent domain, independent of LEF/TCF interactions, suggesting a broader and more complex role in cellular processes beyond its traditional function in the canonical Wnt pathway (Doumpas et al. 2019).

Lastly, β-catenin–activating drugs, such as BC21 (Kahn 2014), may significantly affect fetuses in pregnant patients, requiring careful consideration of their use during pregnancy.

## Author Contributions

R. Mahmoud, contributed to data acquisition, analysis, and interpretation, drafted and critically revised the manuscript; A. Simon, contributed to data acquisition, analysis, and interpretation, critically revised the manuscript; J. Luther, J. Pothe, Y. Du, C. Nottmeier, E. Okine, S. Knauth, M.G. Lopez, contributed to data acquisition and analysis, critically revised the manuscript; E. Bockamp, J. Krivanek, A. LeBlanc, J. Helms, M. Amling, M. Kaucka, T. Schinke, contributed to data interpretation, critically revised the manuscript; T. Koehne, J. Petersen, contributed to conception, design, data analysis and interpretation, drafted and critically revised the manuscript. All authors gave final approval and agree to be accountable for all aspects of the work.

## Supplemental Material

sj-docx-1-jdr-10.1177_00220345251336191 – Supplemental material for Wnt1’s Differential Effects on Craniofacial Bone and Tooth Development Supplemental material, sj-docx-1-jdr-10.1177_00220345251336191 for Wnt1’s Differential Effects on Craniofacial Bone and Tooth Development by R. Mahmoud, A. Simon, J. Luther, J. Pothe, Y. Du, C. Nottmeier, E. Okine, S. Knauth, M.G. Lopez, E. Bockamp, J. Krivanek, A. LeBlanc, J. Helms, M. Amling, M. Kaucka, T. Schinke, T. Koehne and J. Petersen in Journal of Dental Research
