# Supplementary material for: Wnt1’s Differential Effects on Craniofacial Bone and Tooth Development
Source: J Dent Res. 2025 Jun 2;104(12):1415–24. doi: 10.1177/00220345251336191 (PMC12508500; doi:10.1177/00220345251336191)
Supplement: sj-docx-1-jdr-10.1177_00220345251336191 – Supplemental material for Wnt1’s Differential Effects on Craniofacial Bone and Tooth Development [file sj-docx-1-jdr-10.1177_00220345251336191.docx]

*Wnt1's Differential Effects on Craniofacial Bone and Tooth Development*

**Authors:** Ruba Mahmoud, Alexander Simon, Julia Luther, Johannes Pothe, Ying Du, Cita Nottmeier, Erica Okine, Sarah Knauth, Marcos Gonzalez Lopez, Ernesto Bockamp, Jan Krivanek, Aaron LeBlanc, Jill Helms, Michael Amling, Marketa Kaucka, Thorsten Schinke, Till Koehne and Julian Petersen

**Appendix Methods:**

**Lead contact**

Requests for further information should be directed to and will be fulfilled by the lead contact, Julian Petersen (julian.petersen@medizin.uni-leipzig.de).

**Materials availability**

Access to data will be provided to researchers subject to submission of a research proposal and signing a Data Use Agreement. Interested researchers can request access to the data by contacting the corresponding author via E-Mail.

**Data and code availability**

Bulk-population RNA-sequencing datasets generated as part of this study are available at the NCBI Sequence Read Archive (NCBI Accession: GSE267284).

Computational scripts used for genomics analyses conducted as part of this study are available at Github: https://github.com/Ruba-Mahmoud/Bulk-RNAseq-analysis-code-dosage-impact-of-Wnt1.

Any additional information required to reanalyze the data reported in this study is available from the lead contact upon request.

### Mice:

To investigate the role of *Wnt1* in this osteocyte and osteoblast subset, we used an inducible *Wnt1* transgenic mouse model (hereafter called Wnt1Tg), that included a doxycycline (DOX)-dependent Tet-off System. In this model, a promoter-driven tetracycline-controlled transcriptional activator (*Col1a1-tTA*) governs conditional *Wnt1* transgene overexpression in *Col1a1*-expressing cells under the 2.3kb fragment promoter exclusively in osteoblasts, osteocytes, cementoblasts, cementocytes, and odontoblasts (Rossert et al. 1995).

All animal experiments were in accordance with the ARRIVE guidelines (du Sert et al. 2020) and approved by the Landesdirektion Sachsen for animal experiments (TVV 22/21 Zucht). Housing conditions included open or individually ventilated cages in a controlled pathogen-free facility with 12-h light/dark cycle, 65% relative humidity, and 20-24 °C ambient temperature in groups not exceeding 6 animals with wood shavings bedding, nesting material, and access to tap water and standard rodent chow (1328P from Altromin or 1324P doxycycline-containing diet from Altromin with addition of 2.95g/50kg doxycycline hyclate) ad libitum. Furthermore, every effort was taken to ensure the guiding principles of the three R’s (Replacement, Reduction, and Refinement) were followed.

Wnt1Tg mice were generated by breeding Col1a1-rtTA mice (Peng et al. 2008) with ptet-Wnt1 mice (Gunther et al. 2003) on a mixed background. Genotyping tail biopsies were used to distinguish hetero double transgenic mice (Wnt1Tg) and control mice (mice lacking either Col1a1-rtTA or ptet-Wnt1), and all other genotype litters were excluded from the study. Mice at the age of E15.5, P0, and P14 were used with a minimum of biological triplicates, randomly assigned (coin flip), and experiments (micro–computed tomography [CT], and staining) were performed in a blinded manner. Only the corresponding authors were aware of the group allocation.

### Micro-CT

The skulls were prepared and scanned as previously described (Koehne et al. 2013). The skulls were prepared and scanned as previously described (Koehne et al. 2013) using (µCT-40; Scanco Medical) with a voxel size of 7.9 μm, 80 kV, 124 µA, and 800 ms (Koehne et al. 2013). After three-dimensional reconstruction, teeth, mandibles and targeted bone were segmented, and volumes were analyzed, lengths, and angles were calculated using Avizo - 3D image data processing software (FEI, USA).

**Segmentation of the Skulls:**

Reconstructed micro-CT data of skulls were imported into Avizo 3D 2022.2. The voxel size was adjusted to match the scan resolution. The skull was aligned along the axes, and the "Resample Transformed Image" operation was performed. The data set size was reduced by resampling to a voxel size of 20.49 µm, creating a ".resampled" file. Using the Segmentation tab, a ".labels" file was created, and the Threshold function was applied to determine initial and target thresholds. The magic wand tool was used to select a point on the skull, with thresholds adjusted for bone selection. The BounTI (Didziokas et al. 2024) algorithm was run with 50 segments and 55 iterations, using the ".resampled" file as input. The resulting ".Segmentation" and ".Seed" files were refined by merging materials for each bone and using the magic wand and lasso tools to adjust selections. BounTI was run again with the improved seed, repeating the process 1 to 3 times to generate an accurate ".Segmentation" file. Bone materials were named, and surfaces were generated with the "Generate Surface" tool. Surface area and volume were calculated using Avizo.

**Trabecula and Cortical Measurements of the mandible:**

The scan data was imported into Avizo 3D 2022.2 as previously described. Using the "Volume Edit" tool, the left half of the mandible was isolated. The Auto Thresholding tool was then applied to create a ".labels" file. Next, the "Compute Ambient Occlusion" tool was used, followed by the "Interactive Thresholding" tool to select the bone pores. This process was repeated on the pores file to isolate trabecular bone. The "Arithmetic" tool was then used to subtract the trabecular bone from the labels file, generating a cortical bone file (Expression: A-B). A "Segmentation" file containing all three materials (trabecular, cortical, and other bone) was created using the expression: 1A+2B+3*C. The materials were named accordingly, and the "Material Statistics" tool was used to calculate the volume of the trabecular and cortical bones.

**Histology:**

Histology specimens were fixed in 4% PBS-buffered formaldehyde for 24h. E15.5, and P0 pups stayed non-decalcified whereas P14 and P28 mice were decalcified using 10% EDTA for two weeks. Next, jaws were dehydrated in ascending alcohol solution and embedded in methylmethacrylate. 5-μm-thick sections were cut with a Microtec rotation microtome (Techno-Med, Germany). Sections were deparaffinized and stained by tartrate-resistant acid phosphatase (TRAP), Pentachrome, and hematoxylin & eosin staining according to standard protocols. Staining was imaged with a high-resolution inverted microscope 20× objective lens (DMi 8 Leica Microsystems).

### TRAP Quantification:

Osteoclasts quantification was performed using Qupath, where triplicates of Wnt1Tg and control were used. In each sample, 3, and 4 rectangles of the same size were chosen for P0, and P14, respectively, to cover the variance in different jaws and in-between samples. In each rectangle, the bone area was identified and calculated by Qupath. Osteoclasts were counted manually, and the final count was normalized per bone and calculated as the average of all rectangles quantified for the sample.

**Cell culture:**

Osteoclast precursor cells isolated from the bone marrow of femur, tibia, and hip of wildtype mice were plated in a-MEM containing 10% FBS, 1% P/S and 10 nM Vitamin D3. Medium was changed every 2 days. Starting at day 4, Rankl (40 ng/ml), M-CSF (20 ng/ml), and the indicated concentrations of Wnt1/sFRP1 or sFRP1 were added to culture medium. Cells were analyzed at day 8. Cells were cultured in standard incubator conditions (5% CO2, and 37 °C).

**Co-Culture:**

For co-culture, bone marrow cells isolated from femora, tibiae and pelvis of Wnt1Tg and control mice were plated in 24-well plates at a density of 3x10^6^ cells/well in a-MEM containing 10 % FBS and 1 % P/S. At confluency, osteogenic differentiation was induced by addition of 10 mM ß-glycerophosphate and 50 µg/ml ascorbic acid. After seven days of differentiation, 1x10^6^ freshly isolated bone marrow cells of Wnt1Tg and control mice were seeded on top of the osteoblasts. Osteoclast differentiation was induced by 10 nM Vitamin D3 and 100 nM dexamethasone. Cultures were analysed after nine days of osteoclast differentiation. TRAP staining was performed on dried methanol-fixed cells for 20 minutes using a substrate solution (40 mM sodium acetate, 10 mM sodium tartrate, 700 mM naphthol and 1.6 mM fast red violet, pH5).

### RNA-Scope:

### Paraffin-embedded sections were baked at 60°C for 1 hour, followed by deparaffinization in xylene (2×5 min) and rehydration in ethanol (2×2 min). Sections were air-dried on a flat surface for 5 minutes at room temperature. Endogenous peroxidase activity was quenched with 20 µL of hydrogen peroxide per section for 10 minutes at room temperature. Antigen retrieval was performed in 1× target retrieval buffer using a steamer at 75°C for 30 minutes, followed by two quick washes in water, dehydration in 100% ethanol for 3 minutes, and air-drying. Protease digestion was carried out with 20 µL of Protease Plus per section for 30 minutes in a HybEZ oven at 40°C. Wnt1-specific probes (25 µL per section) were hybridized for 30 minutes in the same oven and omitted for negative controls. Signal amplification was performed sequentially with AMP1 and AMP2 (30 minutes each) and AMP3 (15 minutes) in a HybEZ oven at 40°C, with two 2-minute washes in wash buffer between each step. HRP-C1 was applied for 15 minutes at 40°C, followed by washing. Fluorescent detection was achieved using TSA Vivid Fluorophore 570 diluted 1:1000 in TSA buffer for 30 minutes at 40°C. Sections were washed, counterstained with DAPI for 30 seconds, and mounted with Fluoromount.

### Single cell RNA sequencing:

### All dental tissues from the adult mouse incisor (between 2 and 4 months of age) have been isolated and sequenced as described in (Krivanek et al. 2020).

**Total RNA extraction:**

Mandibular jaws were isolated and carefully cleaned; Lysate was obtained by homogenizing an electrical tissue homogenizer, and RNA extraction was carried out using RNeasy® Mini kit (Qiagen) according to manufacturer’s instructions. The concentration of RNA was measured using a NanoDrop ND-1000 system (quality of RNA was measured using High Sensitivity RNA ScreenTape®).

### Library preparation

In Library Preparation Transposon based RNA-seq with NEB depletion was used. 50ng of total RNA were depleted of ribosomal RNA using the NEBNext® rRNA Depletion Kit v2 (NEB) according to the instructions of the manufacturer. Depleted RNA was transcribed using SuperScript IV reverse transcriptase (ThermoFisher) for 2 h at 55°C. After second strand synthesis (TargetAmp kit (Epicentre)) the DNA was tagmented using the Illumina DNA TDE1 Enzyme and Buffer Kits, which fragments DNA and inserts partial sequencing adapter (Nextera) sequences. Final PCR amplification of the libraries was done using KAPA HiFi HotStart Library Amplification Kit with unique dual indexing by IDT® for Illumina Nextera DNA Unique Dual Indexes Sets. The barcoded libraries were purified and quantified using Qubit Fluorometric Quantification (ThermoFischer Scientific). Size distribution of the library DNA was analyzed using the FragmentAnalyzer (Agilent). Sequencing of 2x150 bp was performed with a NovaSeq sequencer (Illumina) according to the instructions of the manufacturer.

Bulk RNA sequencing was performed for 6 (Control I, Control II, Control II, P0.Wnt1 I, P0.Wnt1 II, P0.Wnt1 III), which resulted in 26,833,670 reads (86,5% were mapped confidently to the genome with insert size of 235 bp) for Control I; 28,829,293 reads (85,6% and 145 bp) for Control II; 24,013,591 reads (87,5% and 148 bp) for Control III; 23,654,438 reads (87,4 % and 227 bp) for P0.Wnt1 I; 23,654,438 reads (87.4% and 149 bp) for P0.Wnt1 II; 29,172,046 reads (48,8% and 130 bp) for P0.Wnt1 III.

### Bulk RNAseq Analysis

Paired-end raw data were processed, adapter trimming and quality filtering were done (Stokowy et al. 2014). Ensemble GRCm39, version 105, genome, and its transcriptome were used as a reference. HISAT2 version 2.2.1 was used to build the genome index as well as to map the reads to the genome (Kim et al. 2019). Salmon v.1.7.0, was used for building transcriptome index and gene expression quantification (Patro et al. 2017). Transcript IDs to the Gene IDs table were made and salmon results were imported using tximport version 1.28.0 (Soneson et al. 2016). Unexpressed genes with a total count of less than five were filtered out to reduce the needed time and memory for computation. We identified differentially expressed genes using DESeq2 v.1.40.2 (Love et al. 2014). Hypothesis testing was performed using the likelihood ratio test. P-values obtained by the Wald test were adjusted for multiple testing using the Benjamini and Hochberg methods (Benjamini and Hochberg 1995). Differentially expressed genes were defined as those with log2 fold change less than −1 or greater than +1 and adjusted p-value less than 0.05. annotation was added to Deseq2 using BiomaRt v.2.58.0 (Durinck et al. 2005), lfcshrink from Deseq2 type: ashr v.2.2-65 was used to shrink log-Fold Change (LFC) estimates towards zero using an empirical Bayes procedure (Stephens 2017). Volcano plots were generated using enhanced volcano v.1.18.0 with logFC threshold greater than 1 or less than −1 with P-value less than 0.05 (Blighe et al. 2018). Heatmap data was normalized using the variance stabilizing transformation (VST) function (Anders and Huber 2010). The data was then mapped using complex heatmap v.2.16.0 (Gu et al. 2016) were three types of genes were mapped related to the Wnt canonical pathway coding ligands, receptors, and signal proteins, later average matrix was calculated using limma voom v.3.56.2 (Ritchie et al. 2015). Later enrichGO (Gene Ontology) plotted Gene annotations including top 50 biological processes ordered by the least P.value of shrinked results (Wu et al. 2021), biological processes were clustered in pairwise termism using ggtree v.3.8.2 (Yu 2020) and tidytree v.0.4.6 by submitting shrinked DGE results then Wnt signaling pathway and osteoclast differentiation plotted by pathway view of enrichKEGG analysis results performed by clusterProfiler (Wu et al. 2021) with false discovery rate less than 0.05. Lastly, the common enrichKEGG results between P0 and P14 analysis were plotted using heatplot of Ernrichplot v.1.20.1 (Yu 2023) including the top 5 affected genes with highest absolute value of the shrinked logFC with significant P-value less than 0.05 for P0 and top 10 for P14.

### Statistics

Data is shown as violin plots including the mean for each group and statistical analysis was conducted using t-test or one-way Anova in GraphPad Prism 9. For each experiment including statistical analysis, a minimum of biological triplicates was used. Serial sections were allocated randomly, and experiments (μCT and staining) were performed in a blinded fashion. Only the corresponding authors were aware of the group allocation.

**Appendix figures:**

**
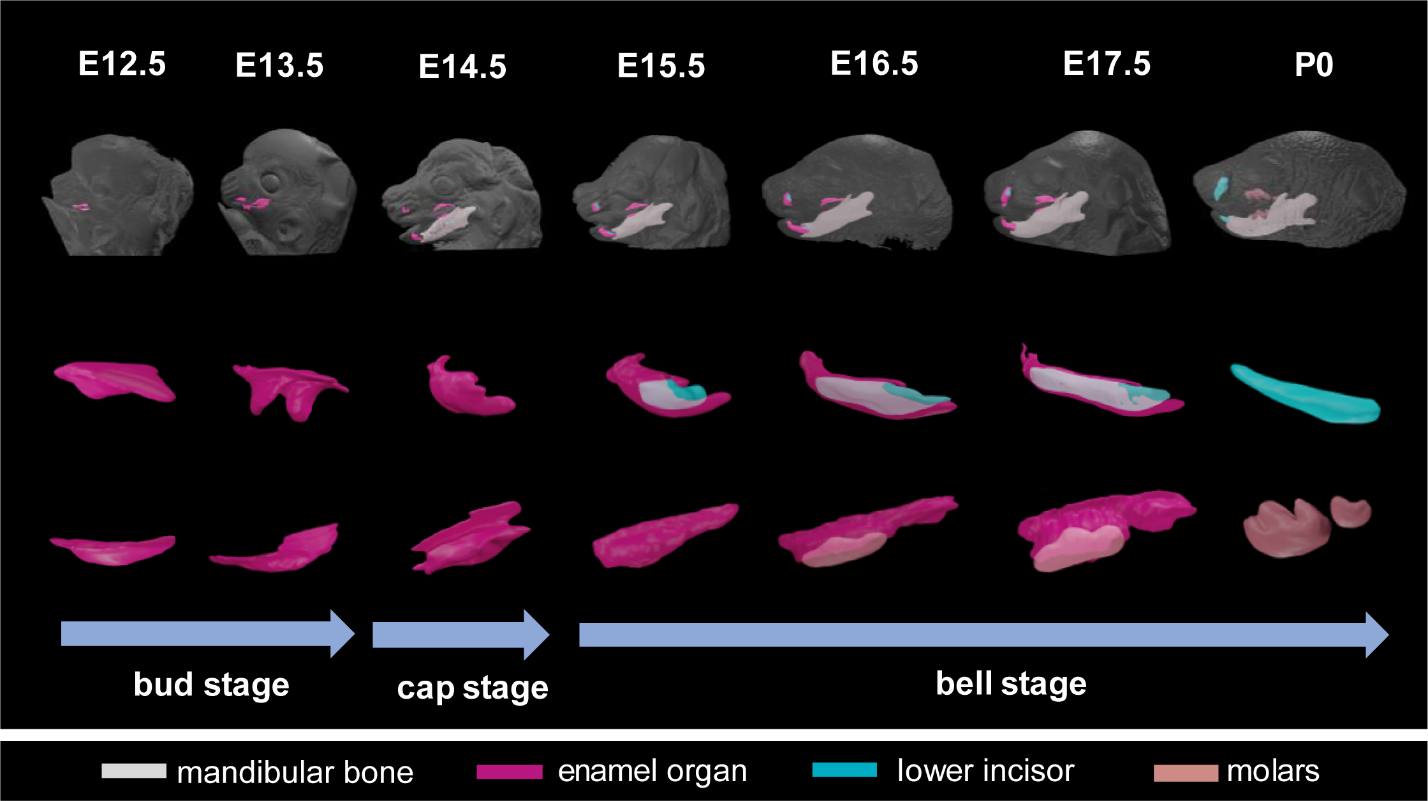
**

**Appendix Figure 1 Natural Micro-CT Mapping Reveals Crucial Timepoints for Tooth Development: E15.5 and P0 Identified as Key Stages**. Embryonic skulls from E12.5 until P0 were mapped using Micro-CT and avizo 3D-segmentation showing the different stages of tooth development starting from bud stage to bell stage and identifying E15.5 where incisor starts developing and P0 as the crown of the first molar is visible alongside the second molar. From day E12.5 onwards, the dental placode of the lower incisors and the molar M1 can be detected. The cap stage began on day E14.5, along with the non-mineralized matrix of the mandibular bone. On day E15.5, the cap-staged teeth had progressed into the bell stage. The dental mesenchyme of the lower incisor thickened, tooth formation proressed, and mandibular bone became denser and assumed its preliminary shape. On day E16.5, M1 can be recognized, followed by M2 at the P0 stage.


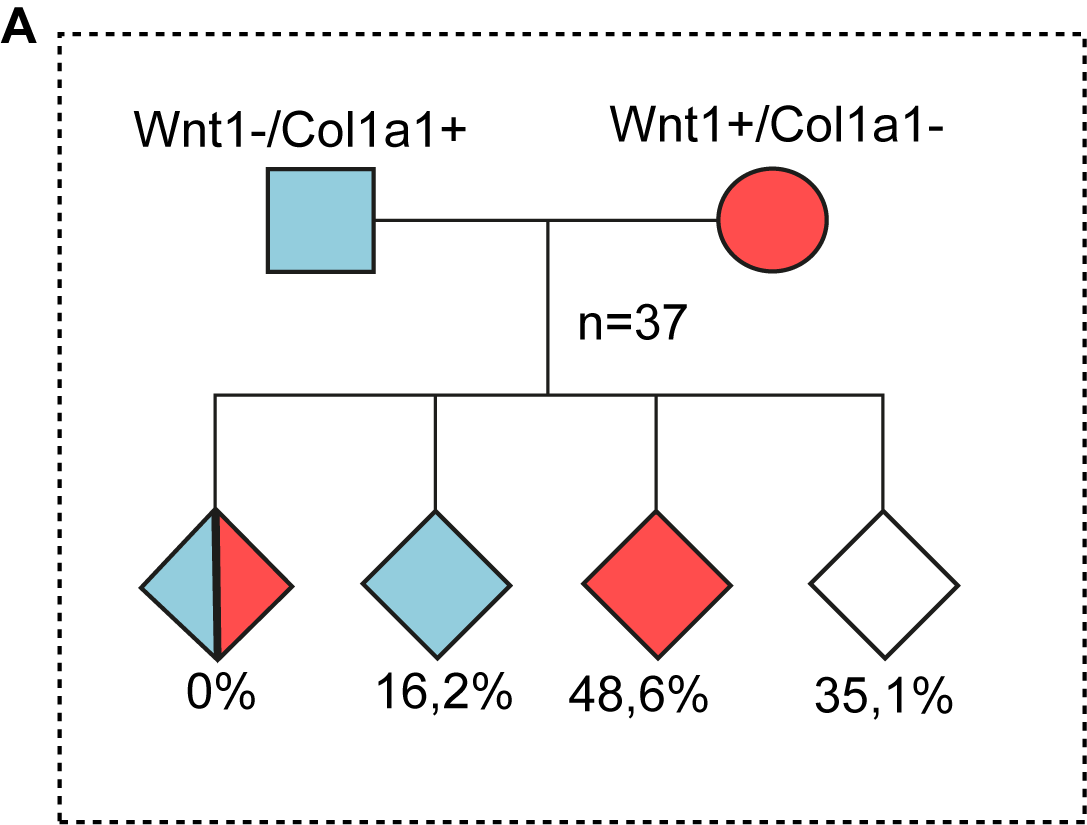


**Appendix Figure 2 Early Wnt1 Activation is Embryonically Lethal and Leads to Spontaneous Abortions:** (**A**) schematic representation showing the Genetic offspring of 37 breeding pairs in the absence of Doxycycline resulted in spontaneous abortions without transgenic pups being born.

**
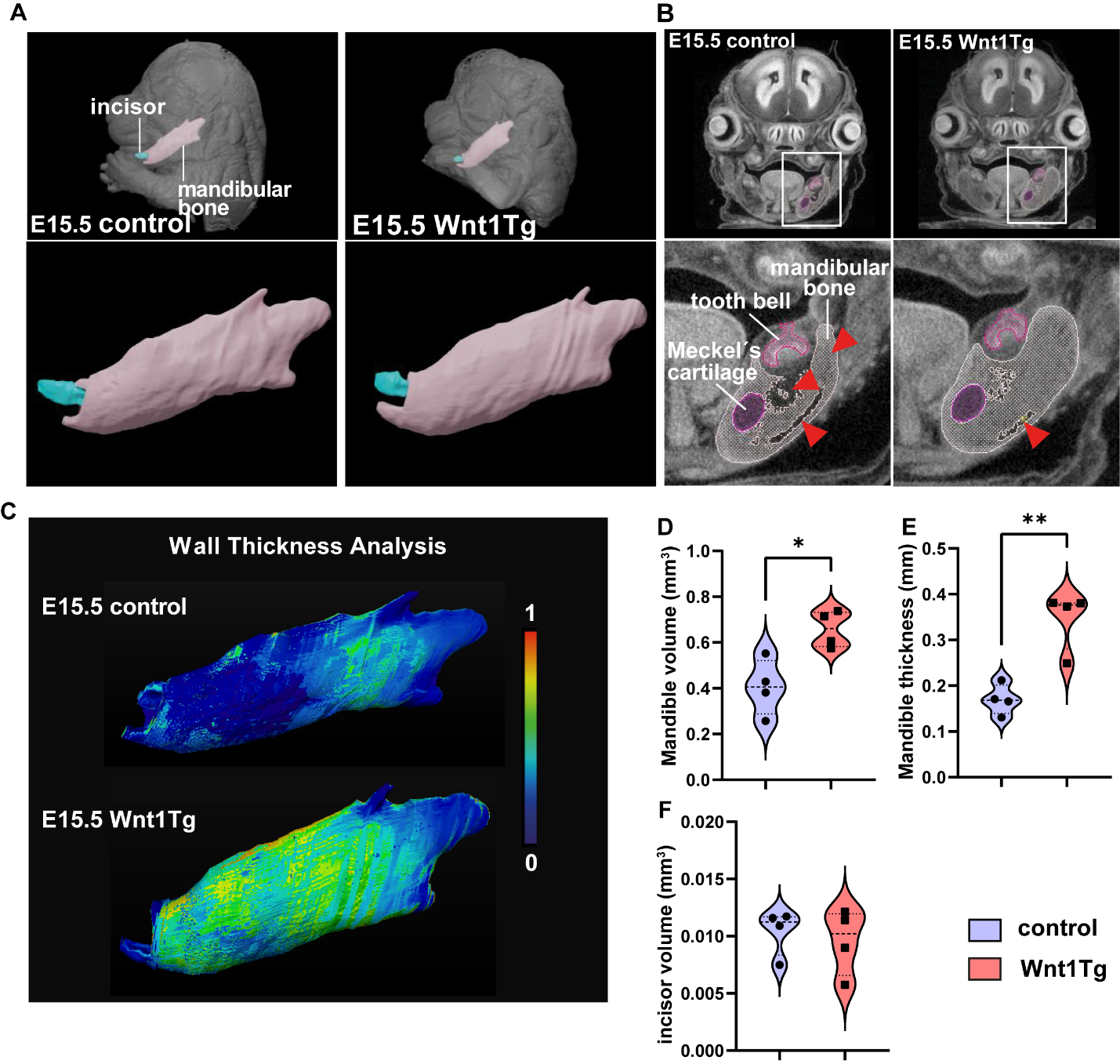
 Appendix Figure 3 Activation of Wnt1 for 5 days Induce Mandible Bone Overgrowth in E15.5 embryos.**

(**A**) Micro-CT and 3-Dimentional images displays overgrowth of the mandible bone in E15.5 Wnt1Tg after 5 days of Wnt1 expression. The volume was determined through 3D segmentation in Avizo. (**B**) Micro-CT coronal sections of E15.5 Wnt1Tg and control of the same mouse. (**C**) Wall thickness analysis of the mandible bone of E15.5 Wnt1Tg and control. (**D-E**) Micro-CT quantification of (**D**) Mandible volume, (**E**) Mandible thickness, (**F**) Incisor volume in E15.5 Wnt1Tg and control embryos. (n=4 P*<0.05, P**<0.01).

**
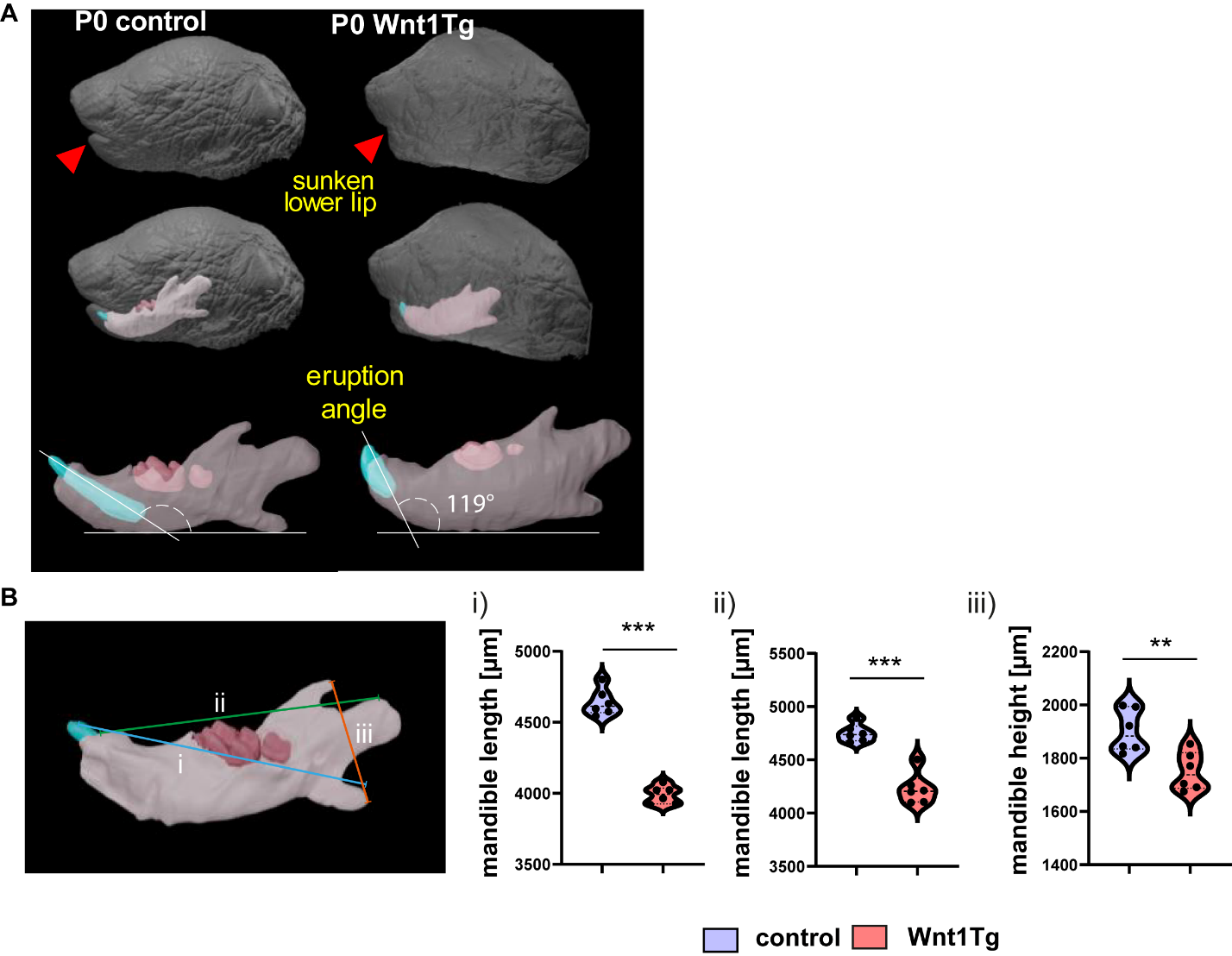
**

**Appendix Figure 4 Micro-CT Analysis and Morphometric Assessment of Skull and Mandible in Wnt1Tg** **Transgenic and Control Pups**

(**A**) Micro-CT images and 3-dimentional images of P0 Wnt1Tg pups and control skulls in addition to lower jaws showing malformation in the skull shape, sunken lower lip, and the eruption angle of the incisor. (**B**) Mandible measurements of control and Wnt1Tg mandibles. From each skull N=3 both mandibles have been measured. P**<0.01, P***<0.001).


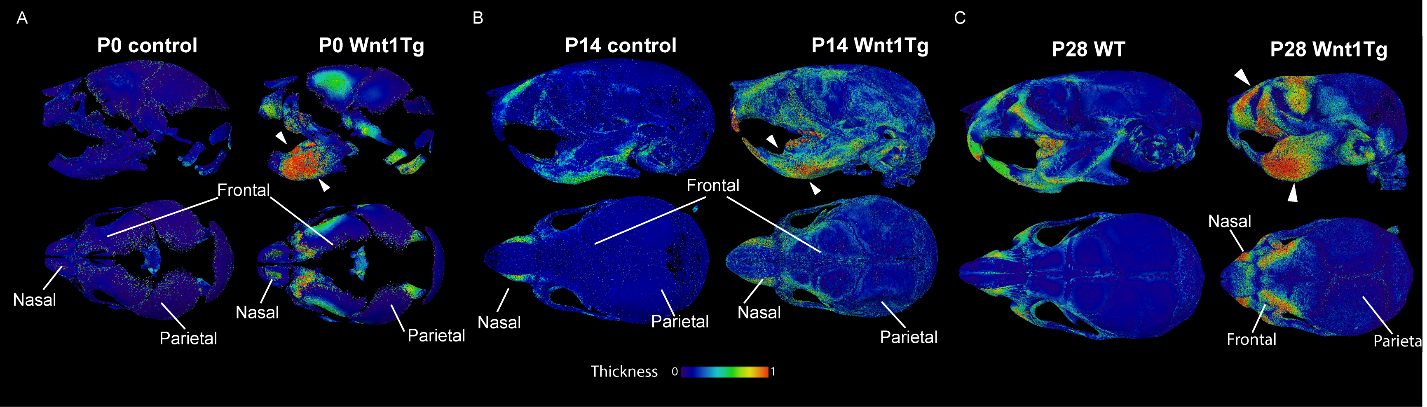
**Appendix Figure 5 Micro-CT Wall Thickness Analysis Reveals Bone Thickness Differences in Mandibular, Nasal, and Frontal Bones of Wnt1Tg** **Transgenic and Control Skulls at P0, P14, and P28**

Wall thickness analysis performed on micro-CT data by 3D Avizo segmentation shows the difference in bone thickness (white arrow) in the mandibular, nasal, and frontal bone in the skull of (**A**) P0 (**B**) P14, and (**C**) P28 Wnt1Tg and controls.

**
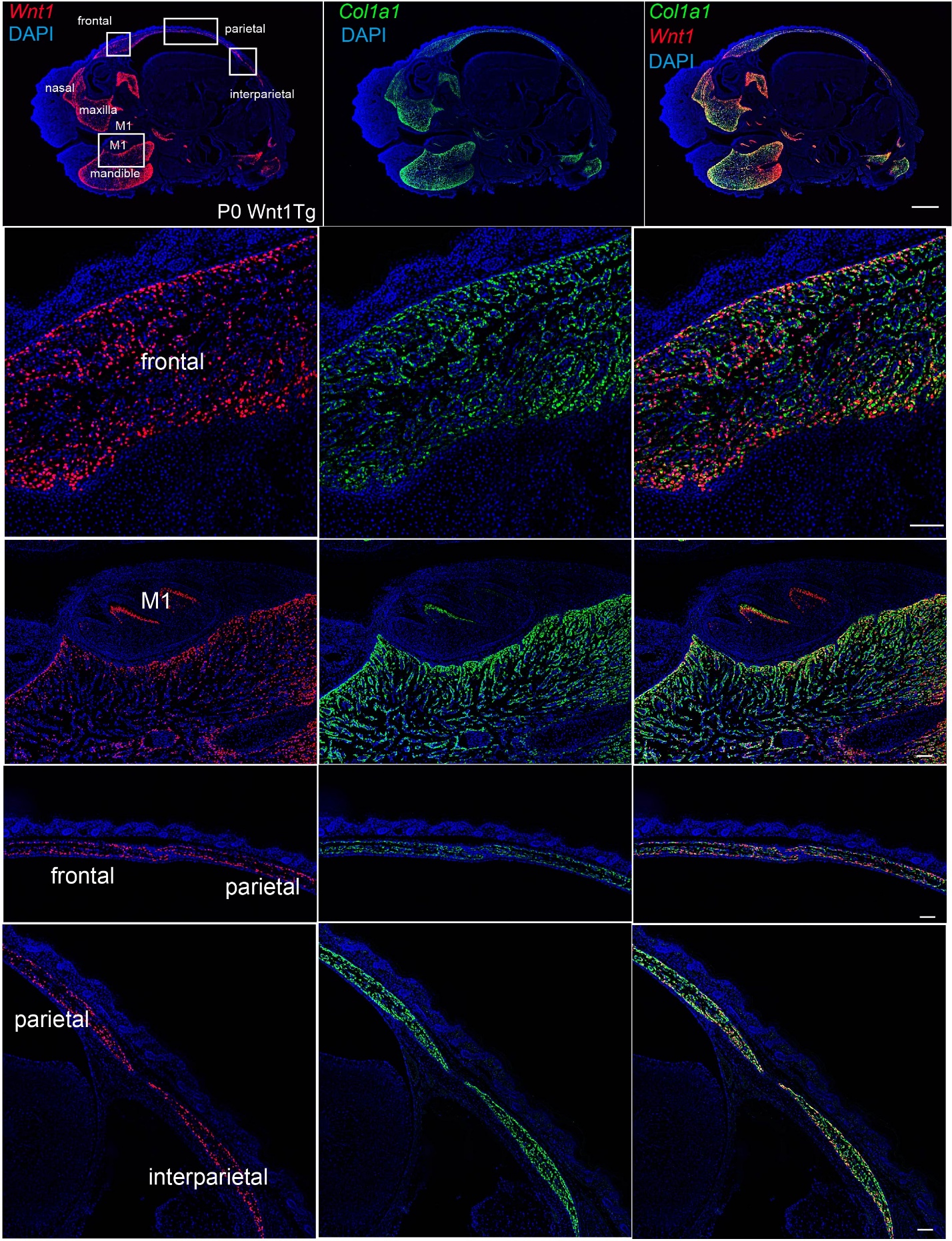
Appendix Figure 6 Expression of Wnt1 and Col1a1 in Craniofacial Bones of P0 Wnt1Tg** **Transgenic Skulls**

In situ hybridization of *Wnt1* and *Col1a1* in a P0 Wnt1Tg skull demonstrates expression in all major craniofacial bones. Scale bars: Overview = 1 mm; Zoomed-in views = 100 µm.

**
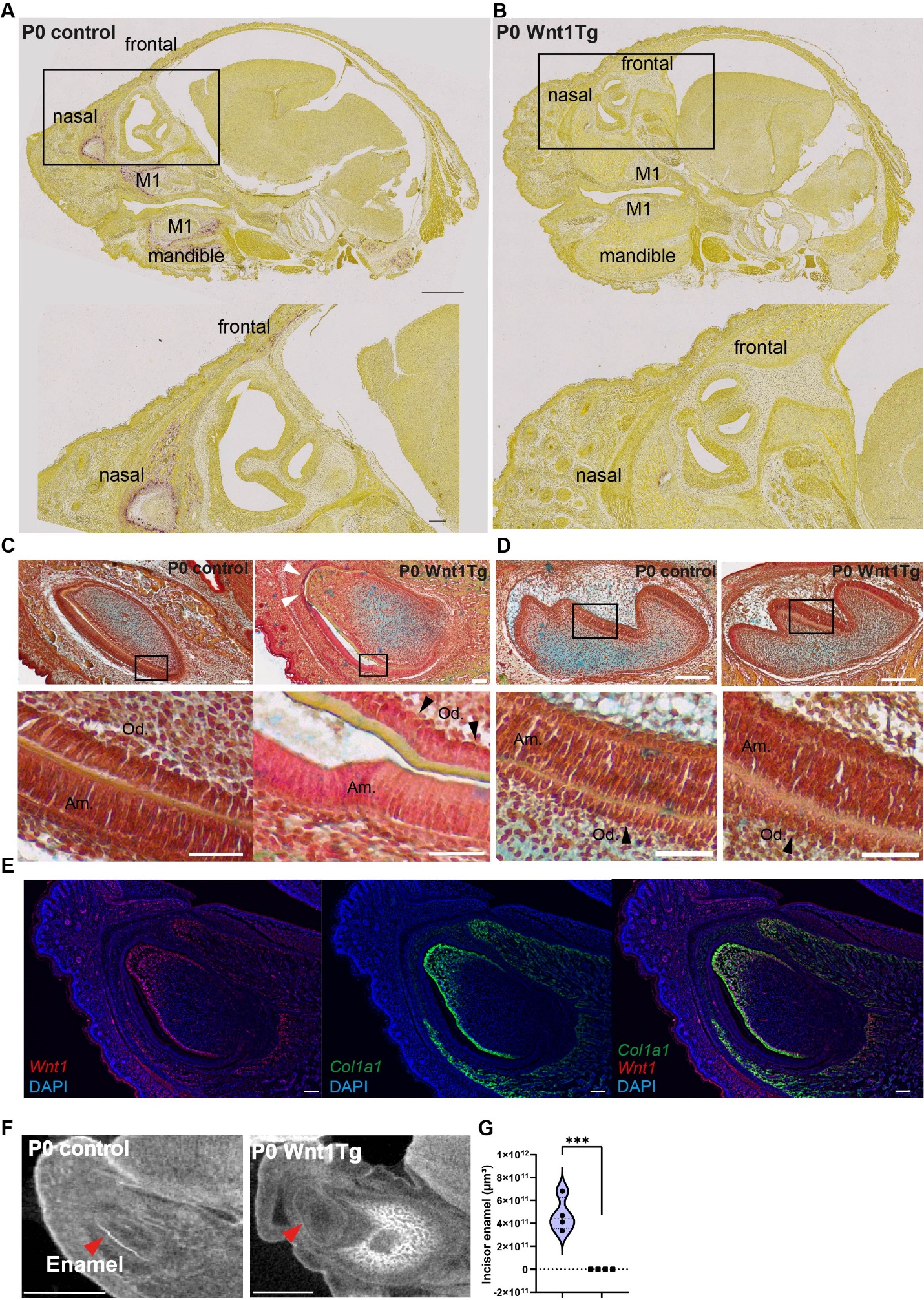
**

**Appendix Figure 7 Histological and µCT Analysis of Craniofacial and Dental Alterations in P0 Wnt1Tg** **Transgenic Mice**

(**A-B**) Overview of representative TRAP staining of (**A**) control and (**B**) P0 Wnt1Tg skulls. Scale bar overview = 1 mm, Scale bar zoom-ins = 200 µm. (**C-D**) Pentachrome staining of the (**C**) mandibular incisor or (**D**) first mandibular molar. Overview scale bar = 200 µm; zoomed-in scale bar = 50 µm. (**E**) In situ hybridization of *Wnt1* and *Col1a1* in the incisor of a P0 Wnt1Tg mouse demonstrates expression of *Wnt1* and *Col1a1* in the alveolar bone as well as odontoblast. Scale bars: 200 µm. (**F**) µCT cross-section of the incisor with a red arrow highlighting the enamel. (**G**) µCT quantification of enamel in the incisor (N=4, P***<0.001).

**
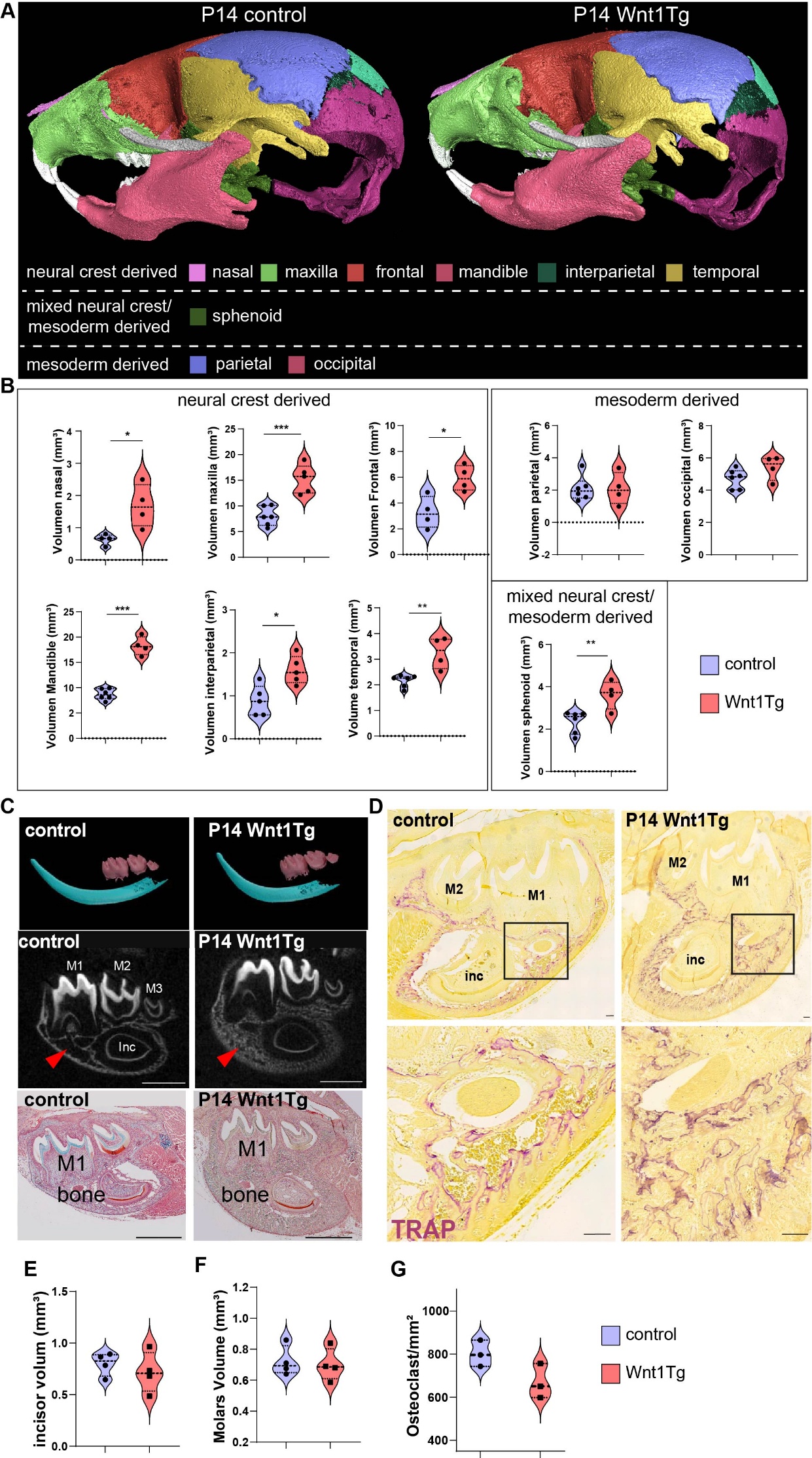
**

**Appendix Figure 8 3D Segmentation, Bone Quantification, and Histological Analysis Highlight Craniofacial and Dental Alterations in P14 Wnt1Tg** **Transgenic Pups**

(**A**) 3D segmentation of all major craniofacial bones of P14 Wnt1Tg pups and control skulls. (**B**) Quantification of all major neural crest, mesoderm and mixed derived bone. (**C**) upper panel: 3D segmentation of mandibular molars and incisor of P14 Wnt1Tg pups and control. Middle panel: Micro-CT sagittal sections and lower panel pentachrome staining of P14 pups showing excessive bone formation (red arrow) scale bar= 1mm. (**D**) Representative images of mandibula TRAP staining in P14 Wnt1Tg and control lower panels show higher magnification of the regions marked by the black rectangles. (Scale bar= 100 µm). (**E-G**) Micro-CT quantification of (**E**) incisor volume, (**F**) Molars volume, and (**G**) osteoclast quantification in TRAP staining in Control and P0 Wnt1Tg pups. (n=4, except for **B** and **G** : n=3, P*<0.05, P**<0.01, P***<0.001).

**
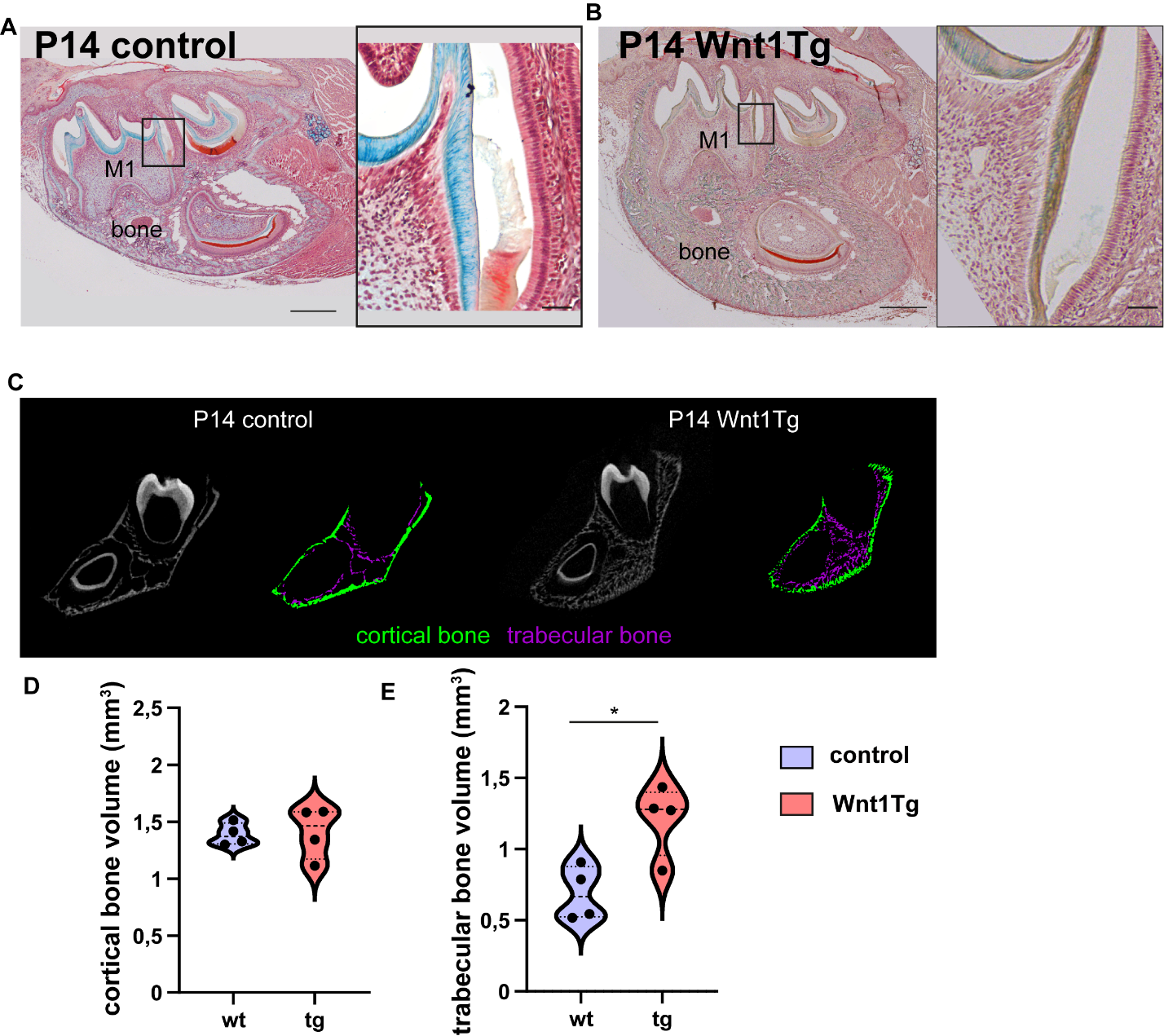
Appendix Figure 9 Histological and Micro-CT Analysis of Cortical and Trabecular Bone in P14 Wnt1Tg** **Transgenic Mandibles**

(**A, B**) Pentachrome staining of control and P14 Wnt1Tg mandibles. Overview scale bar = 0.5 mm; zoomed-in scale bar = 50 µm. (**C**) Representative image of cortical and trabecular bone segmentation performed using Avizo software. (**D**) Micro-CT quantification of cortical mandibular volume and (**E**) trabecular mandibular volume in control and P14 Wnt1Tg mandibles. n=4, P*<0.05

**
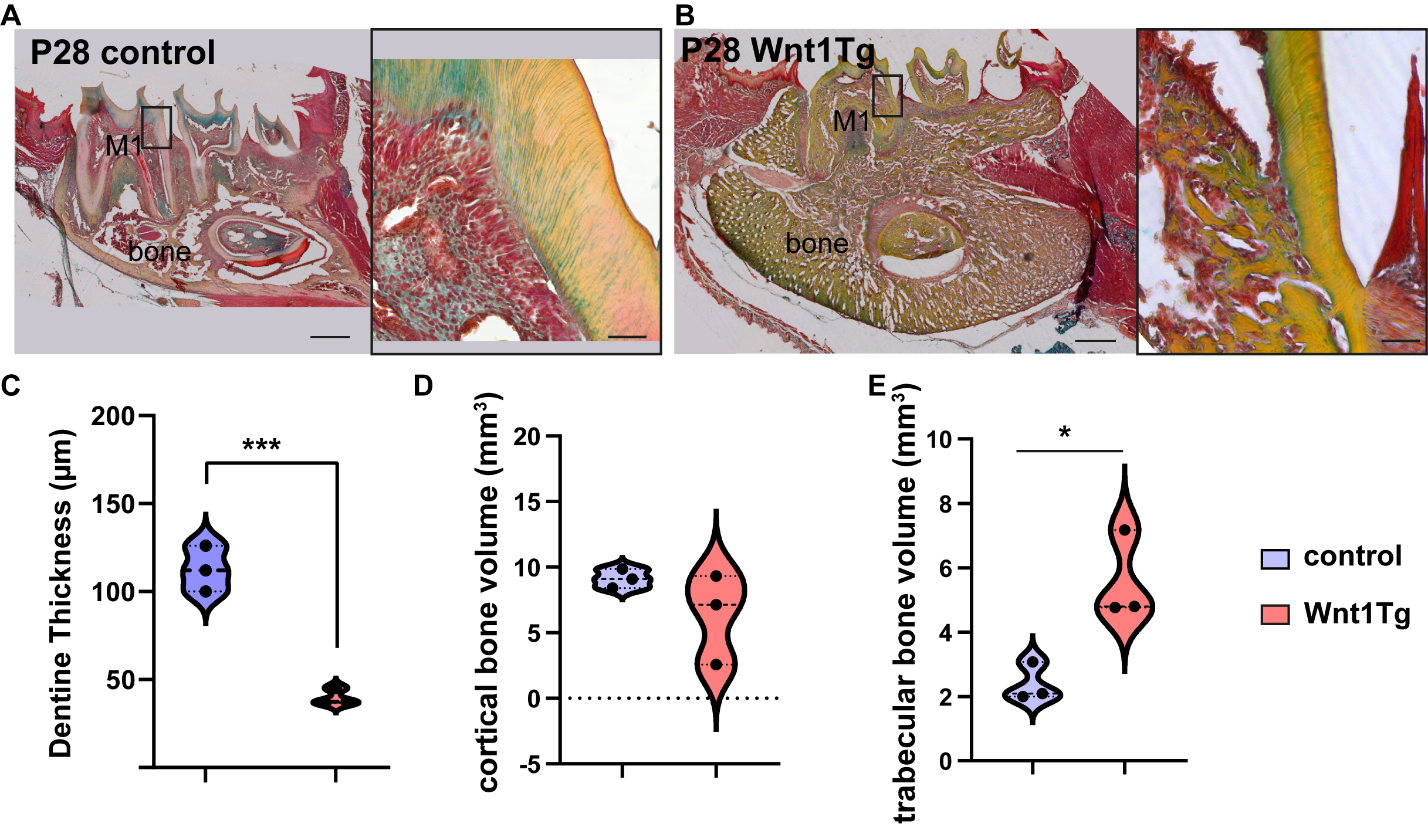
Appendix Figure 10 Histological and Micro-CT Analysis of Cortical and Trabecular Bone in P28 Wnt1Tg** **Transgenic Mandibles**

(**A, B**) Pentachrome staining of control and P28 Wnt1Tg mandibles. Overview scale bar = 0.5 mm; zoomed-in scale bar = 50 µm. (**C**) Quantification of dentine thickness (**D**) micro-CT quantification of cortical mandibular volume and (**E**) trabecular mandibular volume in control and P28 Wnt1Tg mandibles. n=3, P*<0.05, P***<0.001

**
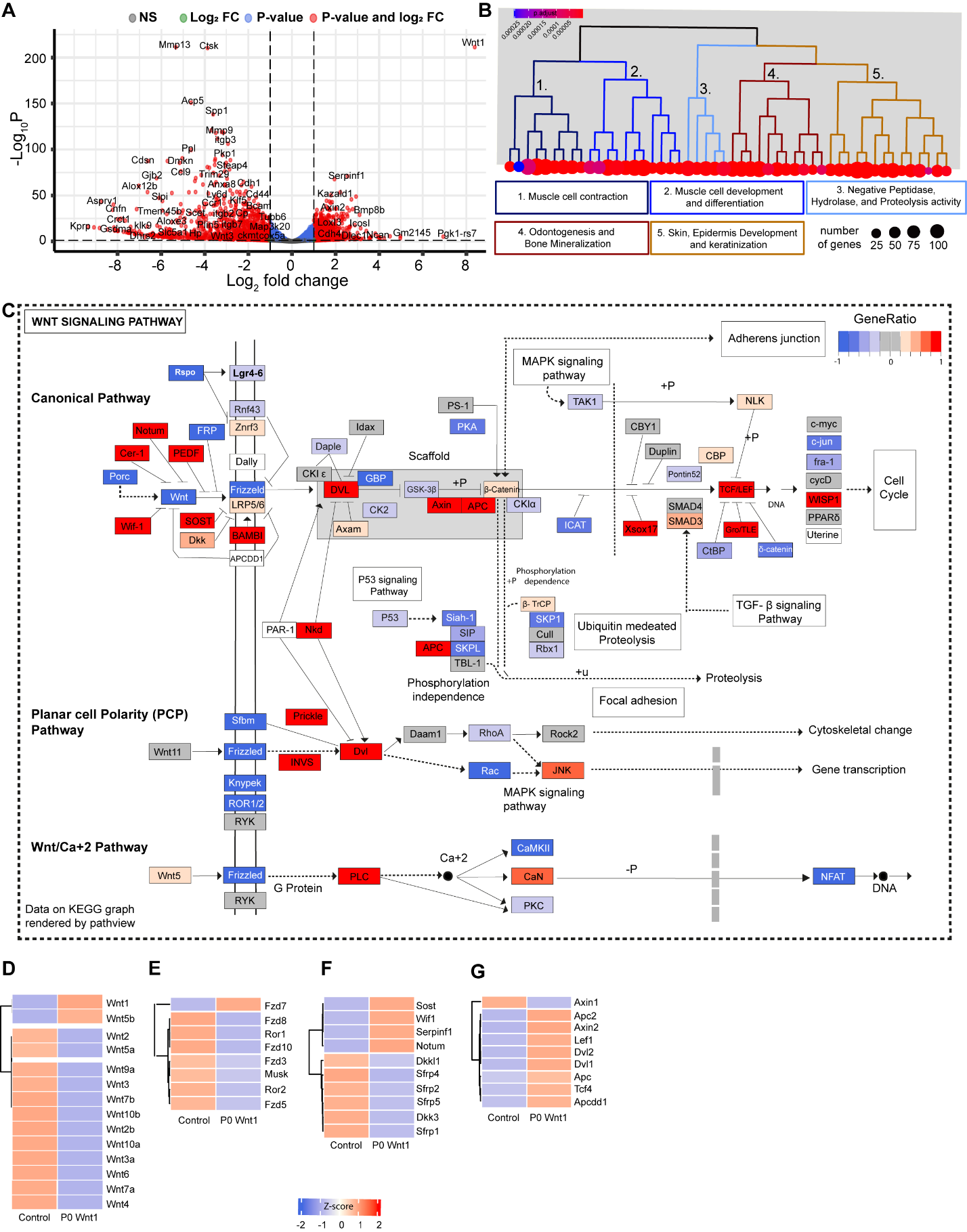
Appendix Figure 11 Bulk RNA-seq and functional enrichment analysis reveals affected genes and related biological process in P0 Wnt1Tg**

(**A**) Volcano Plot showing differentially up and down-regulated genes with |log2fc| > 1 in P0 Wnt1Tg vs control. (**B**) Treeplot highlighting clusters of Gene Ontology (GO) enrichment analysis of the top 50 with the most significant biological processes, we changed clusters labels for simplifying purposes. However, full labelling of plotted biological processes is accessible in **(Appendix Figure 4D)**. (**C**) Pathview of KEGG analysis showing differentially expressed genes associated with Wnt signaling pathway (up-regulated = Red, down-regulated = Blue) in P0 Wnt1Tg versus control. (**D-G**) heat maps of significantly affected ligands (**D**) receptors (**E**) signalling proteins (**F**) and inhibitors (**G**) in the Wnt signaling pathway with z-score between [-2,2].

**
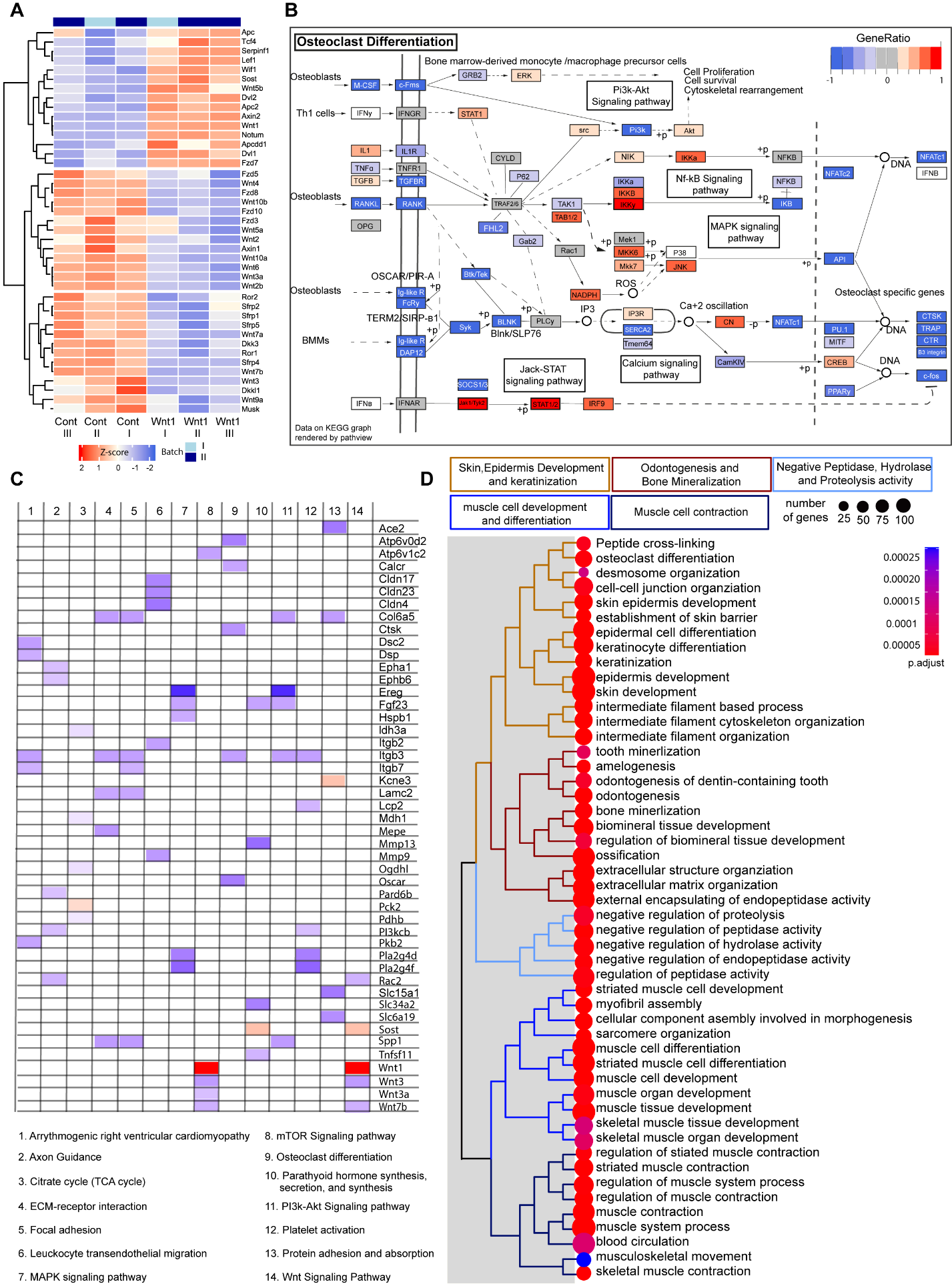
Appendix Figure 12 Bulk RNA-seq analysis in P0 Wnt1Tg** **vs control:** (**A**) heatmap of P0 Wnt1 versus control showing upregulated and downregulated Wnt ligands, canonical Wnt pathway receptors, signaling proteins, and inhibitors in triplicates, according to genotype and batch of sequencing. (**B**) Pathview of KEGG analysis plotting osteoclasts differentiation pathway (up regulated genes = Red, down-regulated genes = Blue). (**C**) Heatplot of the KEGG analysis results showing top 5 affected genes with highest |log2 fold change| in each pathway (up-regulated = red, down-regulated = Blue) common pathways with P14 results were chosen for comparison and the rest according to the pathway importance. (**D**) TreePlot showing clusters of Gene ontology (GO) enrichment analysis with the top 50 significant biological processes, clusters labels were changed for simplifying purposes.

**
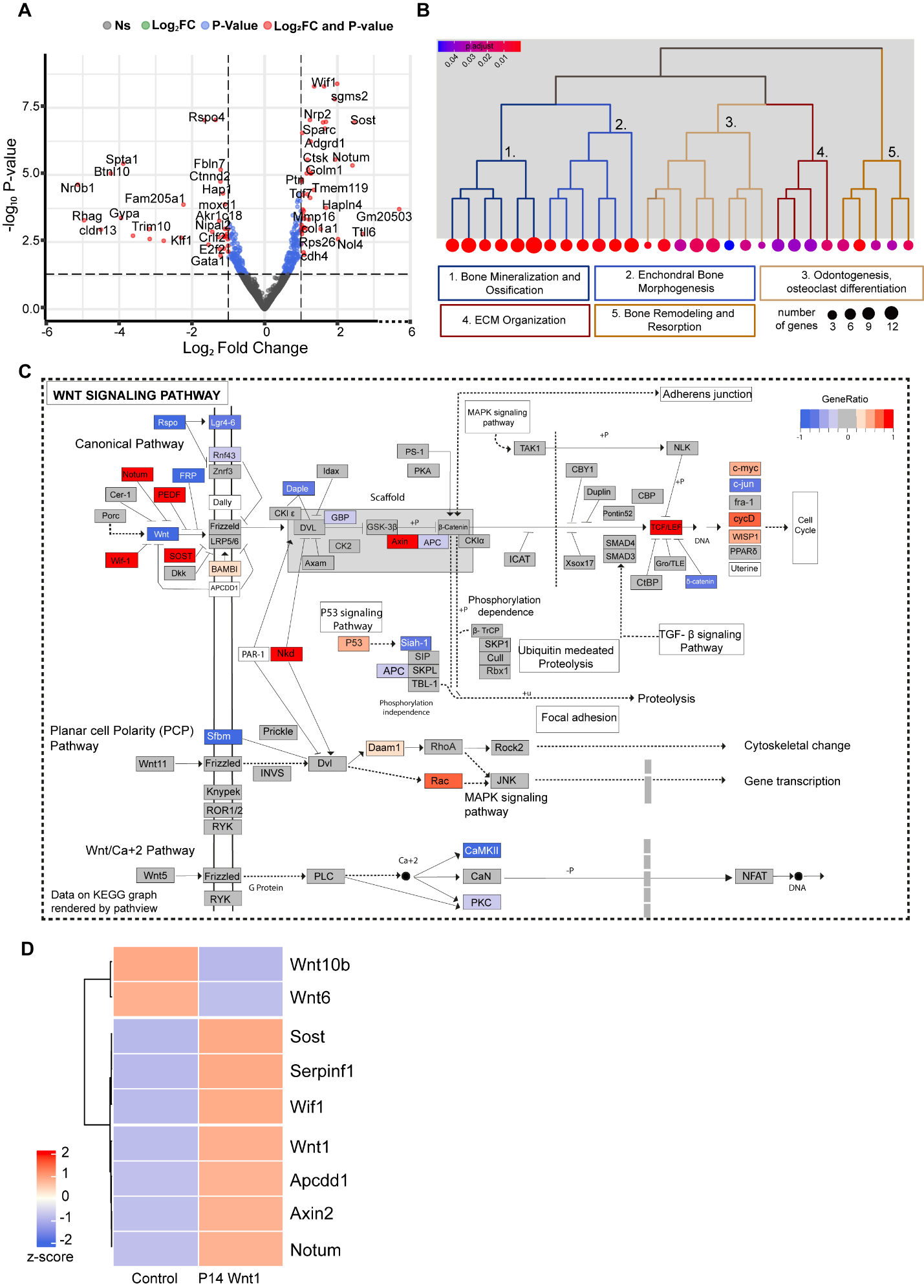
Appendix Figure 13 Bulk RNA-seq and functional enrichment analysis reveals lower activation of Wnt1 accompanied with lower affected genes and related biological process in P14 Wnt1Tg** **vs control in comparison with P0 Wnt1Tg** **vs control**

(**A**) Volcano Plot showing differentially up and down-regulated genes with |log2fc| > 1 in P14 Wnt1 vs control. (**B**) Treeplot highlighting clusters of Gene Ontology (GO) enrichment analysis of all significant biological processes, we changed clusters labels for simplifying purposes. However, full labelling of plotted biological processes is accessible in **(Appendix Figure 6D)**. (**C**) Pathview of KEGG analysis showing differentially expressed genes associated with Wnt signaling pathway (up-regulated = Red, down-regulated = Blue) in P14 Wnt1Tg versus control. (**D**) heat maps of significantly affected ligands, receptors, signalling proteins, and inhibitors in the Wnt signaling pathway with z-score between [-2,2].

**
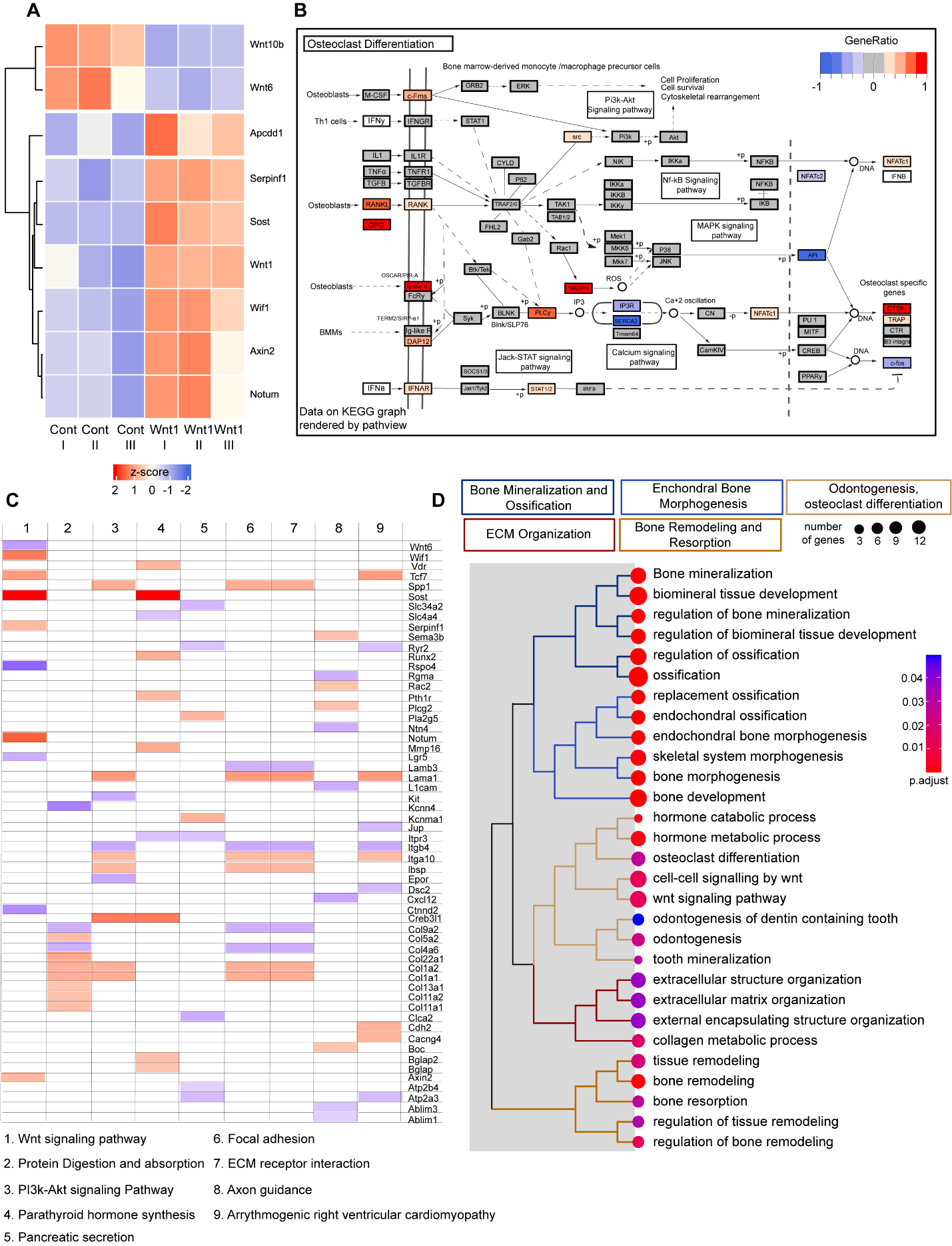
Appendix Figure 14 Bulk RNA-seq analysis in P14 Wnt1Tg** **vs control** (**A**) heatmap of P14 Wnt1Tg versus control showing upregulated and downregulated Wnt ligands, canonical Wnt pathway receptors, signaling proteins, and inhibitors in triplicates, according to genotype and batch of sequencing. (**B**) Pathview of KEGG analysis plotting osteoclasts differentiation pathway (up regulated genes = Red, down-regulated genes = Blue). (**C**) Heatplot of the KEGG analysis results showing top 10 affected genes with highest |log2 fold change| in each pathway (up-regulated = Red, down-regulated = Blue), common pathways with P0 results were chosen for comparison and the rest according to the pathway importance. (**D**) TreePlot showing clusters of Gene Ontology (GO) enrichment analysis of significant biological processes, clusters labels were changed for simplifying purposes.

**
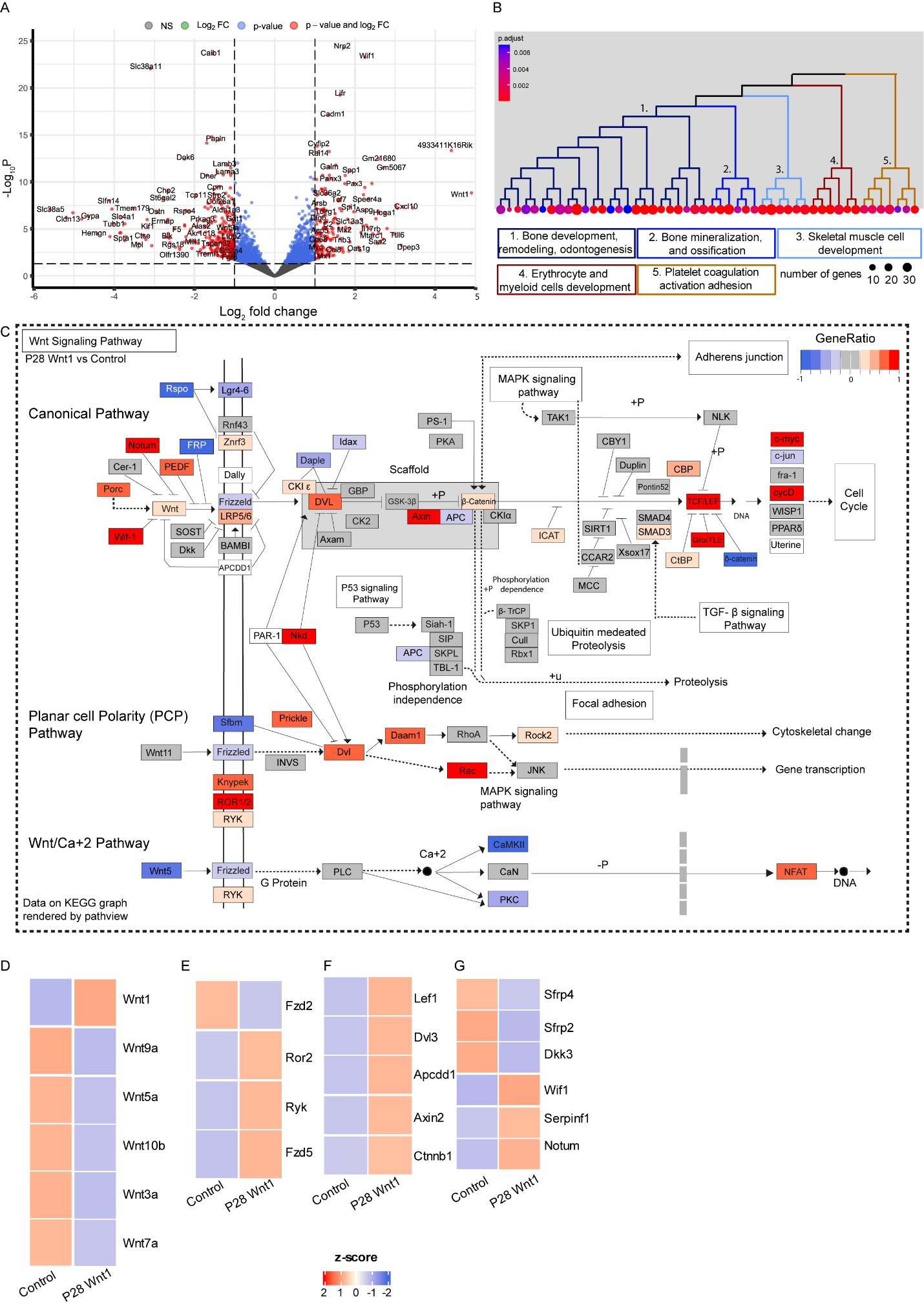
Appendix Figure 15 Bulk RNAseq and Functional Enrichment Analysis Reveal Moderate Gene Expression Alterations in P28 Wnt1Tg** **Mice Compared to P14 Wnt1 and P0 Wnt1 Mice**

(**A**) Volcano plot showing differentially up and down-regulated genes with |log2fc| > 1 in P28 Wnt1Tg vs control. (**B**) Tree plot highlighting clusters of GO enrichment analysis of all significant biological processes, we changed clusters labels for simplifying purposes. However, the full labeling of plotted biological processes is accessible in (**Appendix Figure 16D**). (**C**) Pathview of KEGG analysis showing differentially expressed genes associated with Wnt signaling pathway (up-regulated = red, down-regulated = blue) in P28 Wnt1Tg versus control. (**D-G**) Heat maps of significantly (**D**) affected ligands, (**E**) receptors, (**F**) signaling proteins, and (**G**) inhibitors in the Wnt signaling pathway with z-score between [-2,2] full heatmap of the replicates is accessible (**Appendix Figure 16A**).

**
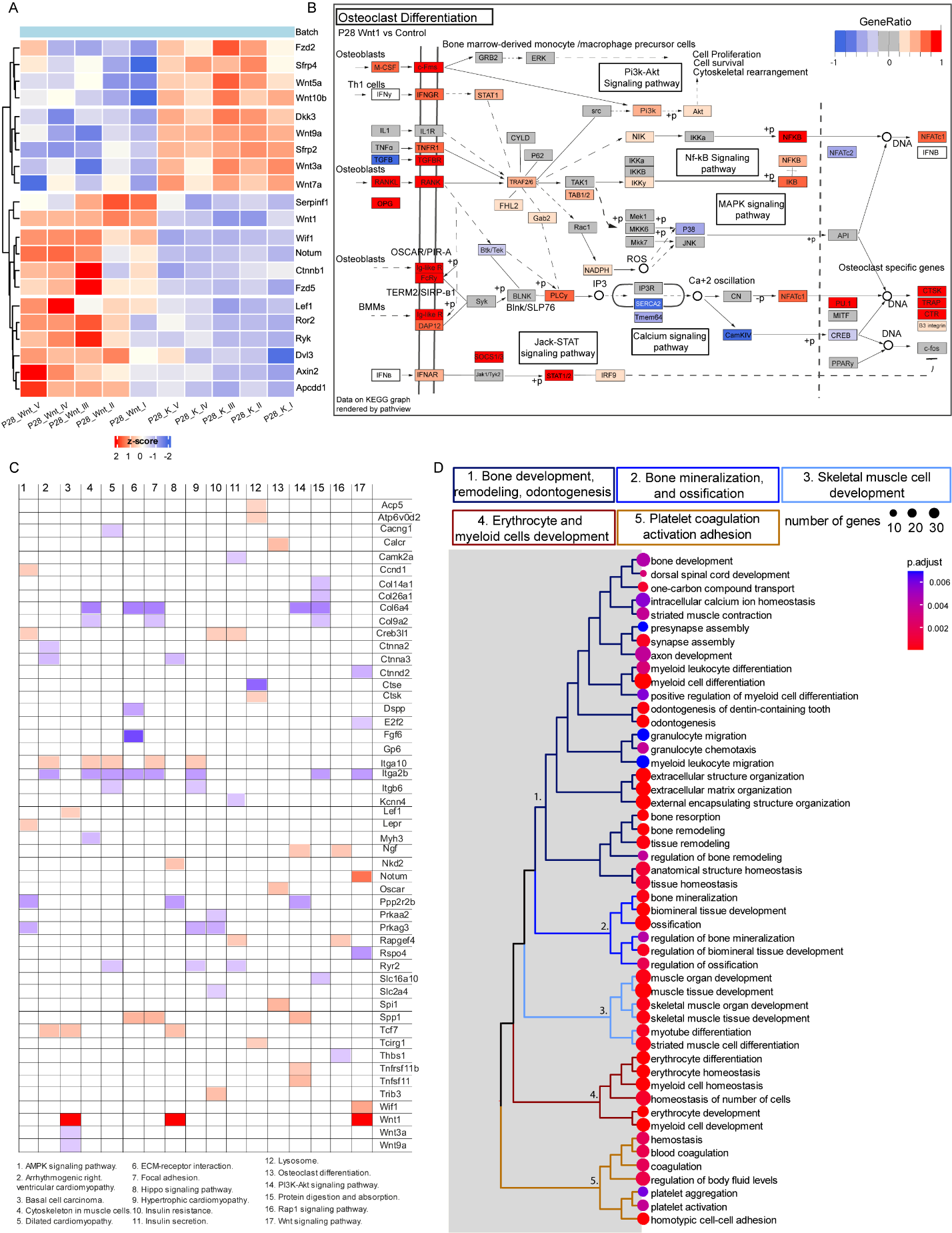
Appendix Figure 16 Figure 4.11 Bulk RNA-seq in P28 Wnt1Tg** **vs control.**

(**A**) Heatmap of P28 Wnt1 versus control showing upregulated and downregulated Wnt ligands, canonical Wnt pathway receptors, signaling proteins, and inhibitors n=5, according to genotype. (**B**) Pathview of KEGG analysis plotting osteoclasts differentiation pathway (up regulated genes = red, down-regulated genes = blue). (**C**) Heatplot of the KEGG analysis results showing top 10 affected genes with highest |log2 fold change| in each pathway (up-regulated = red, down-regulated = blue), common pathways with P0 results were chosen for comparison and the rest according to the pathway importance. (**D**) TreePlot showing clusters of GO enrichment analysis of significant biological processes, clusters labels were changed for simplifying purposes.

**Appendix References:**

Anders S, Huber W. 2010. Differential expression analysis for sequence count data. Genome Biology. 11(10):1-12.

Benjamini Y, Hochberg Y. 1995. Controlling the false discovery rate: A practical and powerful approach to multiple testing. Journal of the Royal Statistical Society: Series B (Methodological). 57(1):289-300.

Blighe K, Rana S, Lewis M. 2018. Enhancedvolcano: Publication-ready volcano plots with enhanced colouring and labeling. Http s. githu b com/kevin bligh e/Enhan cedVo lcano.

Didziokas M, Pauws E, Kolby L, Khonsari RH, Moazen M. 2024. Bounti (boundary-preserving threshold iteration): A user-friendly tool for automatic hard tissue segmentation. J Anat. 245(6):829-841.

du Sert NP, Ahluwalia A, Alam S, Avey MT, Baker M, Browne WJ, Clark A, Cuthill IC, Dirnagl U, Emerson M et al. 2020. Reporting animal research: Explanation and elaboration for the arrive guidelines 2.0. PLoS Biology. 18(7).

Durinck S, Moreau Y, Kasprzyk A, Davis S, De Moor B, Brazma A, Huber W. 2005. Biomart and bioconductor: A powerful link between biological databases and microarray data analysis. Bioinformatics. 21(16):3439-3440.

Gu Z, Eils R, Schlesner M. 2016. Complex heatmaps reveal patterns and correlations in multidimensional genomic data. Bioinformatics. 32(18):2847-2849.

Kim D, Paggi JM, Park C, Bennett C, Salzberg SL. 2019. Graph-based genome alignment and genotyping with hisat2 and hisat-genotype. Nature Biotechnology 2019 37:8. 37(8):907-915.

Koehne T, Marshall RP, Jeschke A, Kahl-Nieke B, Schinke T, Amling M. 2013. Osteopetrosis, osteopetrorickets and hypophosphatemic rickets differentially affect dentin and enamel mineralization. Bone. 53(1):25-33.

Krivanek J, Soldatov RA, Kastriti ME, Chontorotzea T, Herdina AN, Petersen J, Szarowska B, Landova M, Matejova VK, Holla LI et al. 2020. Dental cell type atlas reveals stem and differentiated cell types in mouse and human teeth. Nature Communications 2020 11:1. 11(1):1-18.

Love MI, Huber W, Anders S. 2014. Moderated estimation of fold change and dispersion for rna-seq data with deseq2. Genome Biology. 15(12):1-21.

Patro R, Duggal G, Love MI, Irizarry RA, Kingsford C. 2017. Salmon provides fast and bias-aware quantification of transcript expression. Nature Methods 2017 14:4. 14(4):417-419.

Ritchie ME, Phipson B, Wu D, Hu Y, Law CW, Shi W, Smyth GK. 2015. Limma powers differential expression analyses for rna-sequencing and microarray studies. Nucleic Acids Research. 43(7):e47-e47.

Rossert J, Eberspaecher H, De Crombrugghe B. 1995. Separate cis-acting DNA elements of the mouse pro-alpha 1(i) collagen promoter direct expression of reporter genes to different type i collagen-producing cells in transgenic mice. Journal of Cell Biology. 129(5):1421-1432.

Soneson C, Love MI, Robinson MD. 2016. Differential analyses for rna-seq: Transcript-level estimates improve gene-level inferences. F1000Research. 4.

Stephens M. 2017. False discovery rates: A new deal. Biostatistics. 18(2):275-294.

Stokowy T, Eszlinger M, Świerniak M, Fujarewicz K, Jarza̧b B, Paschke R, Krohn K. 2014. Analysis options for high-throughput sequencing in mirna expression profiling. BMC research notes. 7(1).

Wu T, Hu E, Xu S, Chen M, Guo P, Dai Z, Feng T, Zhou L, Tang W, Zhan L et al. 2021. Clusterprofiler 4.0: A universal enrichment tool for interpreting omics data. Innovation. 2(3).

Yu G. 2020. Using ggtree to visualize data on tree-like structures. Current Protocols in Bioinformatics. 69(1):e96-e96.

Yu G. 2023. Enrichplot: Visualization of functional enrichment result. R package version 1.20. 1.
